# Oxytocin as an Anti-obesity Treatment

**DOI:** 10.3389/fnins.2021.743546

**Published:** 2021-10-13

**Authors:** JingJing Niu, Jenny Tong, James E. Blevins

**Affiliations:** ^1^VA Puget Sound Health Care System, Office of Research and Development, Medical Research Service, Department of Veterans Affairs Puget Sound Health Care System, Seattle, WA, United States; ^2^Division of Metabolism, Endocrinology and Nutrition, Department of Medicine, University of Washington School of Medicine, Seattle, WA, United States

**Keywords:** obesity, food intake, energy expenditure, oxytocin - therapeutic use, adipose tissue

## Abstract

Obesity is a growing health concern, as it increases risk for heart disease, hypertension, type 2 diabetes, cancer, COVID-19 related hospitalizations and mortality. However, current weight loss therapies are often associated with psychiatric or cardiovascular side effects or poor tolerability that limit their long-term use. The hypothalamic neuropeptide, oxytocin (OT), mediates a wide range of physiologic actions, which include reproductive behavior, formation of prosocial behaviors and control of body weight. We and others have shown that OT circumvents leptin resistance and elicits weight loss in diet-induced obese rodents and non-human primates by reducing both food intake and increasing energy expenditure (EE). Chronic intranasal OT also elicits promising effects on weight loss in obese humans. This review evaluates the potential use of OT as a therapeutic strategy to treat obesity in rodents, non-human primates, and humans, and identifies potential mechanisms that mediate this effect.

## Source and Functions of Oxytocin

The obesity epidemic and its associated complications (Eckel et al., 2005; Cornier et al., 2008; Grundy, 2008) increase the risk for cardiovascular disease, cancer, type 2 diabetes (T2D), and death, including that from COVID-19 (Gao et al., 2020; Guo et al., 2020; Jordan et al., 2020; Michalakis and Ilias, 2020; Targher et al., 2020; Zhou et al., 2020) and has become a major health concern (Smyth and Heron, 2006). According to the National Center for Health Statistics, age-adjusted obesity prevalence between 1999–2000 and 2017–2018 has increased in adults from 30.5 to 42.4% (Hales et al., 2020). More than 78 million adults and 12.5 million children and adolescents in the United States are obese (Ogden et al., 2012) and the estimated annual medical cost of obesity is $147 billion in 2008 United States dollars (Finkelstein et al., 2009). Some of the current pharmacologic therapies to treat obesity [i.e., Qsymia (phentermine + topiramate) and Contrave (naltrexone hydrochloride/bupropion hydrochloride)] can worsen sleep disturbance or worsen depression and are not well tolerated (Hollander et al., 2013; Kusminski et al., 2016; Halseth et al., 2018). While major advances in obesity pharmacotherapy have been made with semaglutide, a glucagon-like peptide-1 receptor agonist, which provides significant weight loss of 15% (Wilding et al., 2021), it has been associated with gastrointestinal side effects, including nausea and diarrhea, in overweight and obese adults without diabetes (Rajagopal and Cheskin, 2021). Understanding the unique contributing factors to obesity and designing targeted interventions to lower the disease burden is an urgent need.

While the neurohypophyseal hormone oxytocin (OT) is well recognized for its role in osmoregulation (Verbalis et al., 1995b), prosocial behavior (Striepens et al., 2011; Yamasue et al., 2012) and reproductive behaviors, including lactation (Braude and Mitchell, 1952) and uterine contraction (den Hertog et al., 2001), it is also being tested as a potential therapy to treat post-traumatic stress disorder (van Zuiden et al., 2017; Flanagan et al., 2018), schizophrenia (Striepens et al., 2011; Montag et al., 2012), autism spectrum disorder (Striepens et al., 2011; Yamasue et al., 2012; Miller, 2013; Young and Barrett, 2015), and obesity (Zhang et al., 2013; Lawson et al., 2015; Hsu et al., 2017). This excitement has translated to 535 completed, ongoing, or pending investigations in humans (ClinicalTrials.gov registry, National Institutes of Health). Given the current state of the obesity epidemic and lack of highly effective treatment options, this review focuses on OT as an anti-obesity therapy and mechanisms that contribute to these effects in genetically obese (Kublaoui et al., 2008; Tung et al., 2008; Maejima et al., 2011; Morton et al., 2012; Altirriba et al., 2014) and diet-induced obese (DIO) rodents (Deblon et al., 2011; Maejima et al., 2011, 2017; Zhang and Cai, 2011; Zhang et al., 2011; Morton et al., 2012; Edwards et al., 2021b) as well as in DIO non-human primates (Blevins et al., 2015) and obese humans (Zhang et al., 2013; Lawson et al., 2015; Thienel et al., 2016; Hsu et al., 2017) and assesses the translational and therapeutic potential of OT in humans. Due to the short duration of exposure of OT in the majority of clinical trials, one of the challenges that remains will be to examine the safety, tolerability, and efficacy of chronic intranasal OT use and to identify optimal dosing and frequency of administration to evoke clinically meaningful weight loss in individuals with obesity in the absence of adverse side effects (MacDonald et al., 2011; Tachibana et al., 2013; Zhang et al., 2013; Hsu et al., 2017; Cai et al., 2018). The barriers to the use of chronic treatment include concerns about OT-elicited down-regulation of OT receptors (OTRs) (Insel et al., 1992; Peters et al., 2014; Freeman et al., 2018), the potential for increased anxiety (Peters et al., 2014; Winter et al., 2021), impairments in partner preference (Bales et al., 2013), aggression (Rault et al., 2013), hyponatremia (Bergum et al., 2009; Vallera et al., 2017), adverse cardiovascular effects (Pinder et al., 2002; Vallera et al., 2017; Snider et al., 2019) through interactions with vasopressin receptors (Snider et al., 2019), and feelings of distrust in humans with borderline personality disorder (Bartz et al., 2011) (see Miller, 2013; Leng and Ludwig, 2016a; Leng and Sabatier, 2017, for review).

The majority of central nervous system (CNS) OT is synthesized by magnocellular and parvocellular neurons in the paraventricular nucleus (PVN) as well as the magnocellular neurons in the supraoptic nucleus (SON) in mice (Young et al., 1996; Lein et al., 2007; Blouet et al., 2009; Maejima et al., 2009; Zhang et al., 2011; Sutton et al., 2014), rats (Swaab et al., 1975a,b; Sawchenko and Swanson, 1982; Sawchenko et al., 1984; Rinaman, 1998; Grinevich and Neumann, 2020), non-human primates (Antunes and Zimmerman, 1978; Sofroniew et al., 1981; Kawata and Sano, 1982; Ginsberg et al., 1994; Ragen and Bales, 2013), and humans (Dierickx and Vandesande, 1977; George, 1978; Paulin et al., 1978; Sukhov et al., 1993; Koutcherov et al., 2000) (see Gimpl and Fahrenholz, 2001; Ragen and Bales, 2013, for review). OT is also expressed in magnocellular neurons of the PVN and SON of the prairie vole but it still not clear if OT is expressed in parvocellular PVN neurons or whether OT projections to the nucleus accumbens (NAcc) originate from parvocellular or magnocellular neurons in prairie voles (Ross et al., 2009). In addition to the PVN and SON, OT is found in smaller amounts within magnocellular neurons in the anterior commissural nuclei (mouse) (Castel and Morris, 1988), anterior hypothalamus (mole-rat) (Rosen et al., 2008), accessory nuclei (mouse/rat) (Castel and Morris, 1988; Rinaman, 1998), preoptic area (mole-rat) (Rosen et al., 2008) and periventricular nuclei (mouse) (Castel and Morris, 1988). OT is also expressed in the bed nucleus of the stria terminalis (BNST; mole-rat/rat) (Rinaman, 1998; Rosen et al., 2008), caudal subzona incerta (hamsters) (Vaughan et al., 2011) dorsal hypothalamic area (hamsters) (Shi and Bartness, 2001), mediobasal preoptic area (mouse) (Castel and Morris, 1988), medial amygdala (mole-rat/rat) (Rinaman, 1998; Rosen et al., 2008) and septal region (rat) (Rinaman, 1998).

Mature OT and the carrier neurophysin are processed from the OT/neurophysin 1 prepropeptide (Brownstein et al., 1980) with both being stored in the axon terminals prior to release (Renaud and Bourque, 1991). It appears that the predominant role of neurophysin is to target, package and store OT within secretory granules prior to release (see Gimpl and Fahrenholz, 2001, for review). OT is released both locally from somatodendrites from magnocellular OT neurons in the SON and PVN (Pow and Morris, 1989; Zhang and Cai, 2011; Zhang et al., 2011; Wu et al., 2017) (see Ludwig and Leng, 2006, for review) and distally at axon terminals within the neurohypophysis that originate from magnocellular PVN and SON OT neurons. Recent findings indicate that magnocellular OT neurons also send collateral projections to a number of distal extrahypothalamic sites (Zhang et al., 2021). Magnocellular OT neurons within the PVN appear to send collaterals to the amygdala, caudate putamen and NAcc while those in the SON appear to send collaterals to the caudate putamen, NAcc, piriform cortex and lateral septum (Zhang et al., 2021). In addition, OT is also secreted from axon terminals from parvocellular PVN OT neurons that project to the hypothalamic arcuate nucleus (ARC) (Maejima et al., 2014) (mice), NAcc (Ross et al., 2009, prairie voles)/(Knobloch et al., 2012, rats), midbrain ventral tegmental area (VTA) (Shahrokh et al., 2010), hindbrain parabrachial nucleus (PBN) (Ryan et al., 2017) (mice), dorsal motor nucleus of the vagus (DMV) (Sawchenko and Swanson, 1982; Rinaman, 1998) nucleus tractus solitarius (NTS) (Sawchenko and Swanson, 1982; Rinaman, 1998; Sutton et al., 2014) (rats, mice), and spinal cord (Sawchenko and Swanson, 1982; Sutton et al., 2014) (rats, mice).

The extent to which OT is expressed in outgoing projections from the PVN to hindbrain CNS sites linked to the control of body weight appear to vary based on targets and species. Kirchgessner and Sclafani initially proposed that OT projections from the PVN to hindbrain were important in the control of food intake and body weight based on findings from their lab in which knife cuts that sever PVN-hindbrain projections were found to disrupt OT fibers (Kirchgessner et al., 1988) and result in hyperphagia and obesity (Kirchgessner and Sclafani, 1988). There is a large body of evidence linking hindbrain NTS OTRs in the control of homeostatic food intake (Kirchgessner and Sclafani, 1988; Kirchgessner et al., 1988; Blevins et al., 2003, 2004; Blouet et al., 2009; Baskin et al., 2010; Ho et al., 2014; Ong et al., 2015, 2017; Roberts et al., 2017; Edwards et al., 2021b). Studies have suggested that OT acts, in part, at NTS OTRs, to enhance the responsiveness of gastrointestinal satiation signals (Olson et al., 1991b; Blevins et al., 2003, 2015; Baskin et al., 2010; Ong et al., 2015, 2017) to limit meal size. More recent studies have implicated NTS OTRs in the control of food motivation and feeding reward (Wald et al., 2020). The role of hindbrain OTRs in the control of energy balance is further reviewed in Blevins and Ho (2013), Lawson et al. (2020), and McCormack et al. (2020) (food intake) and Section “What Receptor Populations Mediate Oxytocin’s Effects on Brown Adipose Tissue (BAT) Thermogenesis and Energy Expenditure?”. OT has been found to be expressed in up to (1) 6.3–10.1% of neurons with descending projections from the PVN to the dorsal vagal complex (DVC) (Olson et al., 1992) or (2) 11–16% of neurons with descending projections the PVN to the medulla and spinal cord (Sawchenko and Swanson, 1982) (rats). A recent paper found that OT may be found in a substantially higher proportion of neurons that project to the DVC (28.9%) in rats (Maejima et al., 2019). Current findings indicate that species differences (mice vs. rats) could account for the proportion or location where parvocellular PVN OT neurons project to the NTS. Namely, the majority of PVN OT neurons that project to the NTS appear to the reside within the caudal parvocellular PVN in rats (Rinaman, 1998). One recent study by Sutton et al. (2014) determined that there were few OT projections from the rostral parvocellular PVN that terminated within the NTS and that those that did exist were likely fibers of passage. In addition, those OT neurons that are expressed in the rostral PVN appear to project to spinal cord. These findings raise two questions: (1) Do parvocellular PVN OT neurons located within the caudal parvocellular PVN provide the bulk of OT innervation to the NTS in both mice and rats? (2) Is the parvocellular PVN OT projection to the NTS less dense in the mouse model compared to the rat model? Existing findings at least provide indirect evidence in support of a descending PVN to NTS OT projection in a mouse model (Blouet et al., 2009; Matarazzo et al., 2012; Ryan et al., 2017; Wu et al., 2017) and implicate an important role for OTRs within the caudal hindbrain in the control of body weight through reductions of food intake (homeostatic and hedonic) and increases in BAT thermogenesis or core temperature (as surrogate marker of energy expenditure) in both mice (Blouet et al., 2009; Matarazzo et al., 2012; Ryan et al., 2017; Edwards et al., 2021b) and rats (Baskin and Bastian, 2010; Blevins and Ho, 2013; Ho et al., 2014; Ong et al., 2015, 2017; Roberts et al., 2017; Edwards et al., 2021a).

PVN spinally projecting neurons (SPNs) that express OT appear to be involved with modulating cardiovascular function, stress response, thermoregulation and energy expenditure (via BAT thermogenesis) (see Hallbeck et al., 2001; Nunn et al., 2011, for review). Chemogenetic activation of PVN OT neurons, found to send dense projections to thoracic spinal cord in close proximity to choline acetyltransferase (+) (ChAT; marker of cholinergic neurons) neurons, increased energy expenditure (oxygen consumption), tended to increase BAT temperature (*P* = 0.13) and increased Fos (marker of neuronal activation) within ChAT (+) neurons of the thoracic spinal cord (Sutton et al., 2014) in *Oxytocin-ires-Cre* mice. The proportion of OT found in these PVN SPNs range between 20 and 25% (Cechetto and Saper, 1988) and approximately 40% (Hallbeck et al., 2001) (rats). The lateral parvocellular subdivision contained the highest proportion of PVN OT SPNs (47%), followed by the dorsal parvocellular division (31%) and the medial parvocellular division (24%). It will be helpful to direct future studies to examine the extent to which OTRs within the spinal cord and hindbrain NTS are activated in response to cold and produce overlapping or distinct effects on BAT thermogenesis and energy expenditure.

Data from a combination of pharmacological, microdialysis and/or tract tracing studies suggest that PVN OT neuronal projections to the NAcc are involved with modulating social behavior and feeding reward (Ross et al., 2009; Herisson et al., 2016). Herisson et al. (2016) found that direct injections of OT into the NAcc core reduced intake of palatable sucrose and saccharin solutions, thus providing some of the first evidence linking OTRs within the NAcc core to feeding reward [see Klockars et al., 2015; Lawson et al., 2020 for role of NAcc OTRs in the control of food intake]. Based on the finding by Ross et al. (2009), OT fibers within the NAcc appear to be well conserved in terms of density and distribution in prairie voles, meadow voles, mice and rats. OT has also been found to be expressed in 23% of PVN neurons that project to the NAcc in prairie voles (Ross et al., 2009). In the rat model, the number of fibers (and possibly terminals) found within the NAcc that originate from the PVN appear to vary based on region and if the analysis was done on the side ipsilateral or contralateral to the tracer injection site. Some reports indicate that the NAcc contains only a few OT fibers and/or terminals (Sofroniew, 1983) (not determined if origin was PVN or SON) but other reports that examined only those projections originating from the PVN found there to be up to 50 OT fibers (NAcc core; Knobloch et al., 2012) or >50 OT fibers (NAcc shell; Knobloch et al., 2012) with virtually no projections originating from the SON. These latter findings are consistent with a recent study in mice by Yao et al. (2017), who reported the presence of a low density (∼10–20 fibers) in the NAcc. The reason for the differences in OT fiber density within the NAcc across studies is not clear and may be due, in part, to differences in mouse strain, species and heterogeneity of neuronal projections within a CNS site. A recent study suggests that a subset of these projections may arise from either parvocellular PVN OT neurons or magnocellular PVN or SON neurons. Zhang et al. (2021) determined that a subset of magnocellular OT neurons within the PVN and SON send collateral projections to NAcc. It remains to be determined the extent to which such collateral projections to the NAcc may be important in the control of social behavior and feeding reward.

It is well established that the adiposity signal, leptin, acts, in part, in the ARC to reduce body weight in lean animals by activating anorexigenic [proopiomelanocortin (POMC)] neurons while simultaneously inhibiting orexigenic [neuropeptide Y (NPY)/agouti-related peptide (AGRP)] neurons (see Schwartz et al., 2000; Woods et al., 2000, for review). Several lines of evidence implicate ARC POMC neurons and the endogenous melanocortin 3/4 receptor (MC3R/MC4R) agonist, alpha-melanocyte stimulating hormone (α-MSH; derivative of POMC), as an important component relaying leptin input from the ARC to PVN OT neurons that are positioned to project to the hindbrain and enhance the hindbrain neuronal and satiety response to cholecystokinin (CCK), ultimately resulting in smaller meals (Seeley et al., 1997; Olszewski et al., 2001; Zhang and Felder, 2004; Blevins et al., 2009; Baskin et al., 2010). What remains unclear is the role of a recently identified OT projection from the PVN or SON to the ARC in the control of energy balance and whether this projection is primarily involved with the control of food intake (Maejima et al., 2014; Liao et al., 2020), energy expenditure, or both. Initially studies found that OT administration into the ARC reduced food intake (Maejima et al., 2014). Furthermore, chemogenetic stimulation of OTR-expressing neurons in the ARC reduced food intake and fasting-elicited refeeding in mice (Fenselau et al., 2017) implicating an important role of endogenous OTR signaling within the ARC in the control of food intake. ARC POMC neurons appear to be downstream targets of OT action as POMC neurons express OTRs and OT stimulates cytosolic Ca (2+) from POMC neurons (Maejima et al., 2014). A complementary study by Fenselau reported that ARC OTR-expressing neurons are glutamatergic and that 50% of ARC OTR-expressing neurons expressed POMC (Fenselau et al., 2017). In addition, bath application of OT stimulated the firing rate of ARC OTR-expressing neurons in Oxtr-Cre:tdTomato mice (Fenselau et al., 2017). Through neuroanatomical tracing studies Maejima found that OT was expressed in 29% and 24% of PVN and SON neurons that project to the ARC, respectively (Maejima et al., 2014). In contrast, Liao et al. (2020) indicated that while there was dense OT fiber innervation of the ARC there appeared to be only “some axon terminals in the arcuate hypothalamus nucleus (Arc)” in OxtCre=C; Z/AP double-heterozygous mice. Yao et al. (2017) examined the density of OT fibers that originate from PVN OT neurons and found that OT fibers were found in medium density (>20 fibers) in the ARC in mice. Fenselau also identified that ARC-OTR expressing neurons also project to the PVN. Furthermore, optogenetic stimulation of terminals that arise from ARC-OTR (+) neurons that innervate the PVN result in the suppression of food intake (Fenselau et al., 2017). Future studies will need to determine the extent to which specific ARC-OTR cell populations may regulate both food intake and energy expenditure.

Existing data suggest that OTRs within the VMH are important in both the control of food intake and energy expenditure (see Sabatier et al., 2007; Sabatier et al., 2013; Lawson, 2017, for review). Noble and Klockars both demonstrated that direct injections of OT into the VMH reduced chow intake but had no effect on more palatable saccharin or sucrose solutions (Klockars O. A. et al., 2017) in rats (Noble et al., 2014; Klockars O. A. et al., 2017). These findings link VMH OTRs more so to the control of homeostatic feeding rather than feeding reward. While OT has been recently described in projections from the PVN to the VMH (Nasanbuyan et al., 2018) in mice existing data suggest that OT fibers within the VMH are likely fibers of passage. These findings are also consistent with a recent report by Yao et al. (2017), who reported the presence of low density of OT fibers (∼10–20 fibers) within the VMH originated from the PVN in mice. While OT fibers were detected within regions of the ventrolateral VMH (Liao et al., 2020) that express OTRs in mice (Nasanbuyan et al., 2018). Liao further determined that there were “almost no axon terminals” within the VMH (Liao et al., 2020). Leng et al. (2008) further commented that there was “virtually complete absence of OT-containing fibres in the VMH” and that “The VMH contains very few fibres that show any immunoreactivity for either OT or vasopressin” and that “it is not known whether the few OT fibres there are ‘stray’ axons or dendrites of magnocellular neurons or come from parvocellular neurons of the PVN.” In addition, Leng commented that “So far, there has been no direct evidence of any projection to or synaptic innervation of VMH neurons by OT neurons from the parvocellular region of the PVN” (Leng et al., 2008). Thus, while there does not appear to be data to support a PVN OT projection to the VMH in mice or rats, direct administration of OT into the VMH reduces food intake (Noble et al., 2014; Klockars O. A. et al., 2017) and increases energy expenditure (Noble et al., 2014). Leng and colleagues have postulated that OT, following somatodendritic release from magnocellular OT neurons (within the SON or PVN), could be an important source of endogenous OT that could reach VMH OTRs by diffusion (1) to the ventricles and subsequent transport through cerebrospinal fluid (CSF) or (2) within the brain (Ludwig and Leng, 2006; Sabatier et al., 2007) (see Leng and Ludwig, 2008; Leng et al., 2008; Sabatier et al., 2013; Leng and Sabatier, 2017, for review). The hypothesis that somatodendritic release of OT acts, in part, at VMH OTRs to suppress food intake is particularly attractive as Leng and Sabatier (2017) indicated that dendritic release is “delayed and long-lasting, potentially contributing to post-prandial satiety.” In addition, (1) large amounts of OT are released somatodentrically from magnocellular OT neurons, (2) the SON and PVN are found in close proximity to the VMH, (3) there is robust expression of OTRs within the VMH, and (4) magnocellular OT neurons within the SON and neurons within the VMH are activated in response to food intake (Johnstone et al., 2006). Collectively, these studies have begun to address the potential source of endogenous OT to VMH OTRs and whether this source of endogenous OT to the VMH may also be important in the control of food intake (Leng et al., 2008; see Leng and Ludwig, 2008; Sabatier et al., 2013; Leng and Sabatier, 2017, for review).

The OT projection from the PVN to the VTA has been implicated in the control of social behavior and feeding reward (Mullis et al., 2013; Liu et al., 2020b; Wald et al., 2020) (also see Klockars et al., 2015; Lawson et al., 2020, for additional information on VTA OTRs in the control of food intake). Previous data from Mullis et al. (2013) indicate that direct administration of OT into the VTA reduces consumption of highly palatable 10% sucrose solution. Consistent with these findings, Wald et al. (2020) recently found that OT administration into the VTA reduced bar presses in order to consume palatable sucrose pellets, a finding that suggests OT decreases the willingness to work to obtain sucrose pellets. In addition, they found that VTA administration of OT also reduced food seeking behavior toward palatable chocolate pellets (Wald et al., 2020). Liu et al. (2020b) subsequently provided mechanistic data using *in vivo* fiber photometry to suggest that OT reduces food cue (sucrose)-elicited activation of dopamine neurons within the VTA suggesting that OT may reduce reward intake, in part, through an inhibitory effect on dopamine neurons and their response to rewarding food cues. Eric Krause and colleagues provided neuroanatomical confirmation that a subpopulation of VTA OTR (+) neurons express dopamine (10%) [in addition to glutamate (≈ 44%)], some of which project to the NAcc (Peris et al., 2016). Recent reports indicate there is a direct projection from the PVN to the VTA in rats (Shahrokh et al., 2010) and Liao indicated there were “some branch-like terminals” within the VTA in OxtCre = C; Z/AP double-heterozygous mice (Liao et al., 2020). Yao et al. (2017) examined the density of OT fibers that originate from PVN OT neurons and found that OT fibers were found in medium density (>20 fibers) in VTA. A separate study found that approximately 20% of PVN OT neurons were found to project to the VTA following green retrobead injections into mice expressing tdTomato under control of the *Oxt* promoter (Xiao et al., 2017). Beier extended these findings and found that approximately 6 and 13% of PVN OT neurons synapsed onto VTA dopamine and GABA neurons in DAT-Cre and GAD2-Cre mice, respectively (Beier et al., 2015). These findings are consistent with those from Peris et al. (2016) who demonstrated that approximately 5% of OTR (+) neurons within the VTA co-localized with tyrosine hydroxylase in OTR-Cre mice.

Data from pharmacological and chemogenetic studies implicate a role for the PVN OT projection to the PBN in the control of fluid intake (Ryan et al., 2017). Ryan et al. (2017) demonstrated that PBN OTR (+) neurons were activated by NaCl or water repletion. In addition, chemogenetic activation of PBN OTRs resulted in a suppression of fluid intake (Ryan et al., 2017). Photostimulation of OT terminals within the PBN also resulted in activation of 22% of PBN OTR (+) neurons (Ryan et al., 2017). Ryan further demonstrated that PBN receives direct innervation from PVN OT neurons (Ryan et al., 2017). It is notable that Sutton et al. (2014) demonstrated very little OT fiber innervation within the PBN that originated from the rostral PVN although it might be possible that OT neurons in more caudal regions of the PVN may innervate the PBN more heavily. Collectively, the findings by Ryan demonstrate that OTR-expressing neurons within the PBN are important in the control of fluid homeostasis.

Similar to the VMH, the raphe pallidus is an area that receives virtually little to no innervation from PVN OT neurons despite receiving dense projections from other neuron subtypes within the PVN (Luo et al., 2019). Sutton et al. (2014) reported the existence of few OT terminals within the raphe pallidus. Despite this, Kasahara et al. (2015) have determined that OTR (+) neurons are activated in response to cold and that increased OTR signaling within the rostral raphe pallidus helps restore deficits in response to cold-induced thermogenesis in OT receptor deficient mice (see sections “Does Endogenous Oxytocin Impact Cold-Induced Thermogenesis and Energy Expenditure?” and “What Receptor Populations Mediate Oxytocin’s Effects on Brown Adipose Tissue Thermogenesis and Energy Expenditure?” for additional information).

Data from pharmacological and chemogenetic studies implicate a role for the PVN OT projection to the CeA in the control of fear responses (Knobloch et al., 2012) and food intake (Klockars et al., 2018) (also see Lawson et al., 2020, for additional information on CeA OTRs in the control of food intake). Klockars demonstrated that direct injections of OT into the CeA reduced chow intake but had no effect on more palatable saccharin or sucrose solutions in rats (Klockars et al., 2018) while OT within the basolateral amygdala appeared to reduce intake of both chow and palatable solutions. These findings suggest that OTRs within the CeA may be more involved in the control of homeostatic feeding while those in the basolateral amygdala may participate in both homeostatic and feeding reward. Within the CeA, Yao et al. (2017) examined the density of OT fibers that originate from PVN OT neurons and found a small number of fibers within the CeA (∼0–10 fibers) (mice). In contrast, Liao et al. (2020) identified that there are “many axon terminal branches cover the whole central amygdala region including the central amygdala medial division (CeM), central amygdala lateral division (CeL) and central amygdala capsular part (CeC)” using a Oxt^Cre^/^+^; Z/AP mice. OT fibers from the PVN are also found to innervate the CeA (∼12–36 fibers/side) and medial amygdala (MeA) (∼30–59 fibers/side) in rats (Knobloch et al., 2012). The reasons for the differences in OT fiber density within the CeA between studies are unclear although differences with respect to mouse strain, species, and heterogeneity of neuronal projections within a CNS site may play a role.

Oxytocin is also expressed in peripheral tissues including the heart (rats; Jankowski et al., 1998) and rat and human gastrointestinal (GI) tract (Ohlsson et al., 2006; Welch et al., 2009; Paiva et al., 2021) (including neurons of myenteric and submucosal plexus of enteric nervous system) (Paiva et al., 2021) as well as the islets of Langerhans of the pancreas and Leydig cells of the testes in rats (Paiva et al., 2021), although the stimuli that impact the release of OT within these areas, where it is released and extent to which these peripheral sources of OT contribute to energy balance is not clear.

## Oxytocin Receptor Expression in Central Nervous System Sites Associated With Energy Balance in Rodents, Non-Human Primates and Humans

There is widespread expression of OTRs in CNS sites that are linked to the control of food intake or BAT thermogenesis based on being anatomically positioned to control sympathetic outflow to interscapular BAT (IBAT) to potentially control energy expenditure. There is wide overlap in OTR distribution in mice and rats in areas that include the forebrain hypothalamus [ARC, MPA, suprachiasmatic nucleus, and VMH]/mouse (Gould and Zingg, 2003; Yoshida et al., 2009; Hidema et al., 2016; Fenselau et al., 2017; Ryan et al., 2017)/rat (van Leeuwen et al., 1985; Vaccari et al., 1998; Klockars O. A. et al., 2017)] and basal ganglia [e.g., NAcc and CeA/mouse (Yoshida et al., 2009; Hidema et al., 2016; Ryan et al., 2017)/rat (van Leeuwen et al., 1985; Tribollet et al., 1992; Vaccari et al., 1998)], midbrain VTA [mouse (Peris et al., 2017)/rat (Vaccari et al., 1998)] as well as hindbrain PBN [mouse (Ryan et al., 2017)], rostral medullary raphe (raphe pallidus) [mouse (Yoshida et al., 2009; Kasahara et al., 2015; Sun et al., 2019)], AP [mice (Gould and Zingg, 2003; Yoshida et al., 2009; Ryan et al., 2017)], DMV [mouse (Ryan et al., 2017)/rat (Tribollet et al., 1992; Verbalis et al., 1995a; Vaccari et al., 1998)], NTS [mouse (Gould and Zingg, 2003; Yoshida et al., 2009; Sun et al., 2019)/rat (Verbalis et al., 1995b; Baskin and Bastian, 2010; Ong et al., 2015, 2017)] and spinal cord [mouse (Wrobel et al., 2011)/rat (Reiter et al., 1994)]]. OTRs are also found in the SON and PVN in rats (Yoshimura et al., 1993) and subsequent studies identified OTRs on the somata and dendrites of magnocellular OT neurons in lactating female rats but not in male rats (Freund-Mercier et al., 1994) although it is not yet certain if detectability in the female rats is due, in part, to pre-treatment with the OTR antagonist (Freund-Mercier et al., 1994). These autoreceptors are not found in male rats or in untreated lactating rats and have been proposed to contribute to the feed forward effect of OT on its own release during the milk letdown reflex (Freund-Mercier et al., 1994; Freund-Mercier and Stoeckel, 1995). In contrast to rodents, OTRs appear to have a more restricted distribution in CNS sites linked to energy balance within a variety of non-human primate species (cynomolgus, rhesus macaque, and common marmoset) [NAcc, preoptic area, VMH, DMV, and spinal cord (Boccia et al., 2001; Schorscher-Petcu et al., 2009; Freeman et al., 2014)] and humans (CeA, anterior hypothalamus, MPA, PVN, VMH, AP, NTS and spinal cord) (Loup et al., 1989, 1991; Boccia et al., 2013). OT fibers appear to be in proximity of OTR (+) neurons within the ventrolateral VMH in the mouse (Nasanbuyan et al., 2018) (suggesting the presence of synaptic terminals). Similarly, in the rat, the ventrolateral VMH also expresses OT fibers (Daniels and Flanagan-Cato, 2000; Flanagan-Cato et al., 2001) although there appear to be very few OT fibers elsewhere within the VMH of the rat (Caldwell et al., 1988; Jirikowski et al., 1988; Schumacher et al., 1989, see Leng et al., 2008, for review). As mentioned earlier, others have proposed that OT may reach OTRs by diffusion (see Verbalis et al., 1995a; Verbalis, 1999, for review) from magnocellular OT neurons within the SON (see Leng et al., 2008; Sabatier et al., 2013, for review), through the third ventricle (3V) following dendritic release of OT from the PVN or possibly by axonal release within the VMH (Leng et al., 2008). It is important to note that many of the more recent studies have utilized more advanced and complementary screening tools to assess OTR expression in mice compared to the earlier pharmacological and/or antibody screening tools used to identify OTRs in rats in the 1980s and 1990s. Questions about selectivity and specificity of the antibodies and pharmacological tools used in earlier studies limit our ability to more firmly identify species differences, nonetheless, overlapping patterns of OTR distribution within the basal ganglia, hypothalamus, midbrain, hindbrain and spinal cord implicate potentially important roles of these areas in contributing to the control of food intake (homeostatic and hedonic feeding) and energy expenditure that appear to be well conserved across species. OTRs are also found in peripheral sites that include the GI tract (Qin et al., 2009), nodose ganglion (Welch et al., 2009; Brierley et al., 2021), skeletal muscle (Elabd et al., 2014; Gajdosechova et al., 2014, 2015) and bone (Copland et al., 1999; Colucci et al., 2002; Tamma et al., 2009; Colaianni et al., 2012) in rodents as well as white adipocytes or white adipose tissue in both rodents and humans (Muchmore et al., 1981; Schaffler et al., 2005; Tsuda et al., 2006; Altirriba et al., 2014; Gajdosechova et al., 2014, 2015; Yi et al., 2015; Sun et al., 2019). Recent findings by both Sun et al. (2019) and Yuan et al. (2020) have also reported that OTRs are expressed on brown adipocytes or brown adipose tissue. The potential role OTRs on white and brown adipocytes, the GI tract, vagal sensory afferent nerves, skeletal muscle and bone in contributing to the effects of circulating OT in the control of energy balance, muscle maintenance and bone mass is discussed in Section “How Does Oxytocin Impact Body Composition”.

## Is Oxytocin Effective at Reducing Body Weight in Rodent Models of Obesity?

Previous studies have shown that central, systemic [intraperitoneal (IP), subcutaneous (sc) or intravenous], intraoral (in combination with a proton pump inhibitor) (Maejima et al., 2020) or intranasal administration of OT reduces energy intake, body weight or weight gain in DIO mice and rats (Deblon et al., 2011; Maejima et al., 2011, 2017; Zhang and Cai, 2011; Zhang et al., 2011; Morton et al., 2012; Roberts et al., 2017; Labyb et al., 2018; Seelke et al., 2018; Snider et al., 2019; Edwards et al., 2021b), genetically obese mice and rats [e.g., obese Zucker *fatty* (*fa/fa*) rat, Koletsky (*fak/fak*) rat, *ob/ob*, *db/db and Sim1*^±^ mice] (Kublaoui et al., 2008; Maejima et al., 2009; Morton et al., 2012; Altirriba et al., 2014; Iwasaki et al., 2015; Plante et al., 2015; Balazova et al., 2016), ovariectomized rats (Iwasa et al., 2019b), a rat model of dihydrotestosterone-induced polycystic ovary syndrome (PCOS) (Iwasa et al., 2019a), as well as in DIO rhesus monkeys (Blevins et al., 2015).

**FIGURE 1:**
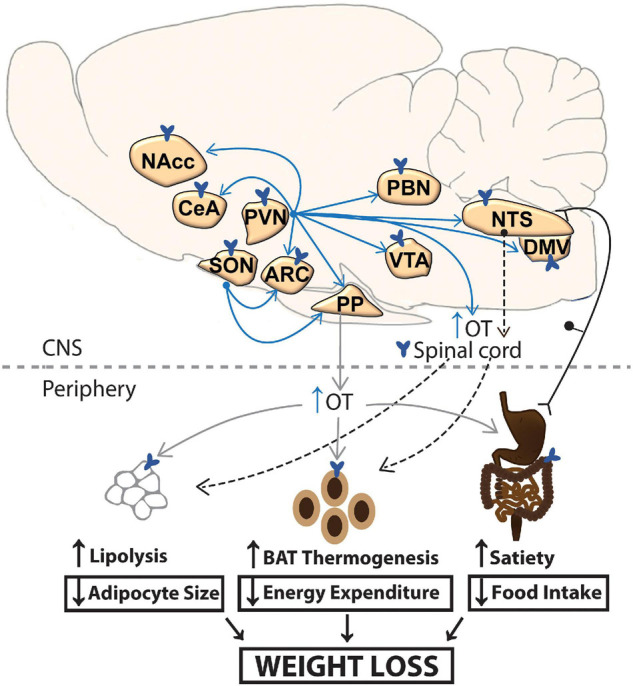
A schematic of circuitry that potentially contributes to the effectiveness of CNS OT on energy homeostasis. OT release within the CNS and spinal cord (shown in blue arrows) or periphery (shown in gray) may impact metabolic processes that result in the reduction of body weight. Dotted arrow represents implicated pathways from NTS to spinal cord and from sympathetic preganglionic neurons in the spinal cord to BAT and WAT. ARC, arcuate nucleus; BAT, brown adipose tissue; CeA, central nucleus of the amygdala; DMV, dorsal nucleus of the vagus; NaCC, nucleus accumbens; NTS, nucleus of the solitary tract; OT, oxytocin; PBN, parabrachial nucleus; PP, posterior pituitary; PVN, paraventricular nucleus; SON, supraoptic nucleus; VTA, ventral tegmental area; WAT, white adipose tissue.

Numerous findings suggest that OT reduces body weight or weight gain in rodents and non-human primates, in part, by reducing energy intake (see Blevins and Baskin, 2015; Lawson et al., 2020; McCormack et al., 2020, for review). It is well documented that OT reduces food intake (including chow, purified low and high fat diet or sucrose solution) following systemic (Arletti et al., 1989, 1990; Deblon et al., 2011; Maejima et al., 2011, 2015, 2017; Morton et al., 2012; Altirriba et al., 2014; Ho et al., 2014; Blevins et al., 2015; Iwasaki et al., 2015, 2019; Balazova et al., 2016; Klockars A. et al., 2017; Iwasa et al., 2019b, 2020; Erdenebayar et al., 2020), intranasal (Maejima et al., 2015) or CNS administration (Arletti et al., 1989, 1990; Olson et al., 1991a; Lokrantz et al., 1997; Rinaman and Rothe, 2002; Kublaoui et al., 2008; Deblon et al., 2011; Morton et al., 2012; Mullis et al., 2013; Ho et al., 2014; Noble et al., 2014; Ong et al., 2015; Herisson et al., 2016; Klockars O. A. et al., 2017; Klockars et al., 2018; Liu et al., 2020a; Edwards et al., 2021a,b). While many such investigations have targeted the lateral ventricles, 3V and 4V, which lack the anatomical resolution to differentiate receptor populations, recent findings indicate that OT reduces food intake following direct injections into the ARC (Maejima et al., 2014), CeA (Klockars et al., 2018), basolateral amygdala (Klockars et al., 2018), VMH (Noble et al., 2014; Klockars O. A. et al., 2017), striatum (NAcc core) (Herisson et al., 2016), midbrain VTA (Mullis et al., 2013) and hindbrain NTS (Ong et al., 2015), many of which express OTRs (see section “Oxytocin Receptor Expression in Central Nervous System Sites Associated With Energy Balance in Rodents, Non-human Primates and Humans”) and are also innervated by OT neurons within the PVN or SON (see Figure 1 and see section “Source and Functions of Oxytocin”). Importantly, OT, at doses that reduce food intake when given into the CNS or periphery, is not associated with increased kaolin diet intake (referred to as pica behavior/readout of visceral illness) (Zhang et al., 2011; Blevins et al., 2016; Roberts et al., 2017; Edwards et al., 2021b) or a conditioned taste aversion test (Zhang et al., 2011; Noble et al., 2014; Iwasaki et al., 2015; Blevins et al., 2016) in lean and obese mice (Zhang et al., 2011; Iwasaki et al., 2014; Roberts et al., 2017; Edwards et al., 2021b) and rats (Noble et al., 2014; Blevins et al., 2016; Roberts et al., 2017). Together, these findings suggest that OT, either given alone, or in combination with other drugs, could be an attractive anti-obesity therapy in DIO and genetically obese rodents and DIO non-human primates. In Section “Does Exogenous Oxytocin Increase Energy Expenditure?,” we will review the potential role of energy expenditure in contributing to the effects of OT on weight loss in rodents and non-human primates.

## Does Exogenous Oxytocin Increase Energy Expenditure?

Numerous studies provide both direct and indirect evidence to indicate that OT is important in the control of energy expenditure. Indirect evidence stemming from pair-feeding studies (amount of food given to vehicle-treated animals is equal to that of OT-treated animals) indicate that OT-treated animals lose more weight relative to pair-fed control animals (Deblon et al., 2011; Altirriba et al., 2014; Blevins et al., 2016). These findings were evident following chronic lateral ventricular infusions of OT (16 nmol/day) in high fat diet-fed male rats (weeks 5 to 7 of high fat diet feeding) or chronic sc infusions of OT (50 nmol/day) into male high fat diet-fed rats (weeks 5 to 7 of high fat diet, 45% kcal from fat) (Deblon et al., 2011), male lean standard diet-fed rats (Deblon et al., 2011) as well as in male *ob/ob* mice (Altirriba et al., 2014). Note that it is not clear the extent to which the rats used in the study by Deblon et al. (2011) were DIO without having the body weight and body composition data pre- and post-dietary intervention and they were only maintained on the high fat diet for a relatively short period of time (5 to 7 weeks). However, similar findings have been obtained following a single acute injection of OT (Morton et al., 2012) in male low-fat diet-fed (10% kcal from fat) or high fat diet-fed (45% kcal from fat) Sprague Dawley rats after having been maintained on the respective diets for ∼4 months. These findings suggest that, in addition to reductions of food intake, other mechanisms (including energy expenditure) also contribute to OT-elicited weight loss. In addition, OT infusions over a 14-day period were found to reduce body weight gain despite no changes in cumulative 14-day food intake (Deblon et al., 2011). Furthermore, the findings from long-term administration studies suggest that OT appears to become less effective at reducing food intake despite an unimpaired and persistent reduction of body weight gain or body weight over this period of time (Deblon et al., 2011; Maejima et al., 2011, 2017; Altirriba et al., 2014; Blevins et al., 2016; Roberts et al., 2017).

The most direct evidence in support of an important role for OT in the control of energy expenditure stems from pharmacological studies that included measurements of energy expenditure. Acute administration of OT into the 3V or VMH boosted energy expenditure or oxygen consumption as determined by indirect calorimetry in rodents (Zhang and Cai, 2011; Zhang et al., 2011; Noble et al., 2014). These effects were recapitulated following peripheral administration in a translational DIO non-human primate (rhesus monkey) model (Blevins et al., 2015). In addition, other paradigms in which OT was administered in a paradigm that elicited weight loss, OT was not found to elevate energy expenditure (Blevins et al., 2016). One explanation for these findings might be that chronic administration of OT could be important in attenuating the counter-regulatory mechanisms that result in weight regain in the setting of prolonged weight loss. Thus, OT, in the setting of weight loss, might prevent the drop in energy expenditure that accompanies prolonged weight loss and restore levels of energy expenditure to that of control animals (Blevins et al., 2016) and mice (Maejima et al., 2011). It is important to acknowledge that energy expenditure was not measured throughout the extent of the treatment period across the chronic treatment studies. We have previously shown that chronic 3V infusions of OT stimulate IBAT temperature during a time that coincides with OT-elicited weight loss (days 2–3 of infusion period) (Roberts et al., 2017) and that 3V OT appeared to maintain IBAT temperature to that of control animals for the remainder of the infusion period (unpublished findings). In addition, following minipump removal and throughout the 4-week washout period, IBAT temperature appeared to be slightly lower in rats that had been previously treated with chronic 3V OT relative to vehicle treated control rats (unpublished findings). It is possible that timing of energy expenditure in relation to OT-elicited weight loss is important and that chronic administration of OT may stimulate BAT thermogenesis and energy expenditure at the onset of OT-elicited weight loss and function, in part, to help maintain weight loss by preventing a drop in BAT thermogenesis and energy expenditure (Blevins et al., 2016) that accompanies prolonged reductions of food intake and weight loss in animals (Fosgerau et al., 2014) and humans (Rosenbaum et al., 2002, 2005, 2010; Schwartz and Doucet, 2010). Current studies are underway to determine the extent to which SNS innervation of BAT is required for OT to increase energy expenditure and elicit weight loss.

### Does Exogenous Oxytocin Increase Brown Adipose Tissue Thermogenesis and Browning of White Adipose Tissue?

We know from recent studies that acute forebrain (3V) and hindbrain (4V) injections of OT stimulate IBAT temperature [functional measure of BAT thermogenesis (Song et al., 2008; Leitner and Bartness, 2009; Vaughan et al., 2011)] in both chow-fed and DIO rats and mice (Roberts et al., 2017; Edwards et al., 2021b). In addition, chronic infusions of OT into the 3V stimulates IBAT temperature at the start of OT-elicited reductions of body weight in DIO rats (Roberts et al., 2017) raising the possibility that BAT thermogenesis might contribute to weight loss in response to OT treatment. OT injections into the midbrain (median raphe) or 4V also stimulated core temperature in mice (Yoshida et al., 2009) and rats (Ong et al., 2017). These findings shed light on the potential contribution of OT in stimulating BAT temperature to help maintain body temperature (Cannon and Nedergaard, 2004) particularly during cold stress (Kasahara et al., 2007, 2013, 2015; Xi et al., 2017). In addition to maintaining core temperature during cold challenges, changes in IBAT temperature are often found to precede and contribute to changes in core temperature under conditions of fever and stress (Kataoka et al., 2014).

The majority of studies indicate that the effects of OT on IBAT temperature appear to contribute to non-shivering thermogenesis (mediated by BAT thermogenesis) rather than shivering thermogenesis (generated by movement of skeletal muscle). Namely, chronic administration of OT is not associated with elevations in locomotor activity in rats (Deblon et al., 2011; Blevins et al., 2016; Iwasa et al., 2019b) or mice (Maejima et al., 2011). Data from Carson et al. (2010) indicate that peripheral administration of OT attenuated methamphetamine-elicited increases in locomotor activity in rats. In addition, peripheral administration of OT decreased locomotor activity in rats; central administration of an OTR antagonist also blocked this effect (Angioni et al., 2016). Furthermore, central administration of OT blocked the ability of central delivery of an OT receptor antagonist to stimulate locomotor activity. However, two findings raise the possibility that increased locomotor activity may contribute, in part, to the elevated IBAT temperature. Sutton et al. (2014) found that DREADD-elicited stimulation of PVN OT neurons in *Oxytocin-ires-Cre* mice was associated with a small elevation of locomotor activity, energy expenditure and sc IBAT temperature and a close to significant elevation of sc IBAT temperature (*P* = 0.13). Another study found that VMH administration of OT stimulated short-term physical activity in rats for 1-h post-injection but these effects failed to coincide with the more prolonged effects of 3V or 4V OT on IBAT temperature that we have found in our studies in rats (Roberts et al., 2017) and mice (Roberts et al., 2017; Edwards et al., 2021b). Yuan et al. (2020) recently reported that OT may stimulate markers of thermogenesis in skeletal muscle [including uncoupling protein-3 (UCP-3)] and further work will need to be determined as to what role this mechanism plays in contributing to the effects of OT on energy balance given that OT has been found to have (1) no impact on locomotor activity, (2) reduce locomotor activity, or (3) produce only short-term changes in physical activity that do not coincide with the temporal profile of OT on IBAT temperature.

Previous studies indicate that OT may help stimulate the transformation of white adipocytes to more metabolically active “brown” adipocytes. The process of “browning” (Nedergaard and Cannon, 2014) of WAT may involve the transdifferentiation or *de novo* synthesis of brown adipocytes in white adipose tissue (WAT) culminating with increased expression of uncoupling protein-1 (UCP-1) and the production of heat (Cannon and Nedergaard, 2004). We recently demonstrated that hindbrain (4V) infusions of OT (16 nmol/day) elicit browning of inguinal white adipose tissue (IWAT) (as indicated by increased UCP-1 expression) in the IWAT of chow-fed mice (Edwards et al., 2021b) but not in DIO mice. In addition, chronic sc OT infusions (125 ng/kg/h or ≈ 66.2 nmol/day) also appeared to stimulate UCP-1 expression in sc fat of *db/db* mice (Plante et al., 2015) but the UCP-1 staining was not quantified. A more recent study by Yuan et al. (2020) reported that chronic sc OT infusions (100 nmol/day) increased UCP-1 expression in IWAT but not in epididymal WAT (EWAT) of high fat diet-fed mice. It is not clear why Yuan found elevated expression of UCP-1 in IWAT of high fat diet-fed mice while we did not find a significant effect of 4V OT to increase UCP-1 in IWAT from DIO mice in our study. It is important to note that the dose used in Yuan’s study in DIO mice was approximately 6.25-fold higher than that found to be effective following 4V infusions in our study and that higher doses may be required to elicit “browning” of IWAT in DIO mice relative to chow-fed mice. It is difficult to compare across studies as chow-fed mice were also not examined in Yuan’s study. Given the existence of outgoing polysynaptic projections from the PVN OT neurons to IWAT (Shi and Bartness, 2001) and EWAT (Shi and Bartness, 2001; Stanley et al., 2010), it is possible that OT acts locally at hindbrain or spinal cord OTRs to elicit browning. In addition, OT may also act peripherally to induce browning of WAT through a direct action on OTRs found on adipocytes (Muchmore et al., 1981; Schaffler et al., 2005; Tsuda et al., 2006; Deblon et al., 2011; Yi et al., 2015) in either IWAT (Gajdosechova et al., 2015) or EWAT depots (Muchmore et al., 1981; Altirriba et al., 2014; Gajdosechova et al., 2014, 2015). Sun et al. (2019) recently found that OT may suppress browning, when it is applied directly to adipocytes *in vitro* as indicated by reduced expression of brown adipocyte specific markers (Cox7a, Cox8b, Cebpb, Retn, and Cidea). Further *in vitro* studies should also include UCP-1 which was not examined in this study. It will be important to determine if the conflicting data are due, in part, to dose, route of administration, acute vs. chronic application, *in vitro* vs. *in vivo* conditions, lack of overlap of brown adipocyte specific markers between studies and if these effects can be blocked by an OTR antagonist. These findings raise the possibility that OT may stimulate energy expenditure through multiple CNS and/or peripheral sites and raise the question as to the extent to which BAT thermogenesis and “browning” of WAT contribute to these effects.

### Does Endogenous Oxytocin Impact Cold-Induced Thermogenesis and Energy Expenditure?

Oxytocin receptor or OT deficient mice are associated with adult-onset obesity (Kasahara et al., 2007; Takayanagi et al., 2008; Camerino, 2009; Sun et al., 2019) that appears at 8 (Tamma et al., 2009), 10 (Kasahara et al., 2007) or 16 weeks (Camerino, 2009) in OT null mice and 12 weeks in OTR null mice (Takayanagi et al., 2008). The adult-onset obesity in the OT and OTR null mice is characterized by increased body weight (Kasahara et al., 2007; Takayanagi et al., 2008; Camerino, 2009), fat mass (Sun et al., 2019) and/or fat pad weight (Takayanagi et al., 2008; Camerino, 2009). The finding that this occurs despite having no changes in overall daily food intake (Kasahara et al., 2007; Takayanagi et al., 2008; Camerino, 2009) suggests that other mechanisms (such as impairments in energy expenditure) may contribute to their obesity phenotype. Daily food intake in OT or OT receptor deficient mice is normal regardless of whether the mice were fed chow (Amico et al., 2005; Takayanagi et al., 2008; Camerino, 2009), sucrose-enriched chow (Amico et al., 2005) or high fat diet (Takayanagi et al., 2008). These findings were confirmed in both chow-fed and high fat diet-fed mice with diphtheria toxin-elicited reductions of PVN and SON OT neurons (Wu et al., 2012; Xi et al., 2017). While one other study also reported that OTR deficient mice do not have any overall changes in daily food intake, meal pattern analysis revealed that they have increased meal size during the dark cycle (Yamashita et al., 2013). However, these effects were offset by no change in meal size during the light cycle as well as no change in meal frequency during the light or dark cycle. Collectively, the findings provide strong evidence that the obese phenotype observed in the OT or OT receptor deficient mice can’t be explained by impairments in daily food intake.

The obesity phenotype observed in older OT and OTR deficient mice does not appear in younger mice. For example, there is no difference in body weight between OTR null mice and wild-type mice that are younger [6–7 weeks (Welch et al., 2014) or 10 weeks (Takayanagi et al., 2008)]. While Sun et al. (2019) also found no difference in body weight between 12-week-old OTR null mice and wild-type mice, the OTR null mice did have increased fat mass raising the possibility that differences in body weight may have been observed at a subsequent time. Camerino (2009) also found no difference in body weight between OT null and wild type mice at 8 weeks. It is not clear if differences in background strain, housing conditions and/or thermoregulation may have contributed to the differences in obesity phenotype at specific ages between studies.

One factor that seems contrary to the adult-onset obesity phenotype observed at 8, 10 or 16 weeks in OT null mice is that finding that OT null mice tend to have a reduction in muscle regeneration by 12 weeks of age and a significant defect in muscle regeneration and a reduction of both muscle mass and fiber size by 52 weeks of age (Elabd et al., 2014) (characteristic of sarcopenia). These mice tended to have an increased number of adipocytes near the site of muscle regeneration (non-significant) but the hindlimb perimuscular and intramuscular adipose tissue deposition of OT null mice was significantly increased relative to wild-type mice (Elabd et al., 2014). While body weight and body adiposity levels were not reported in these particular mice it is possible that the increase in fat mass compensates for any reduction in lean mass to help maintain the obesity phenotype as OT null mice age and this may help to explain, in part, why the obesity phenotype is not observed in younger OT null mice.

Consistent with a physiological role of endogenous OT in the control of thermogenesis, cold has been found to activate PVN OT neurons (Kasahara et al., 2007) and rostral raphe pallidus OTR (+) cells (Kasahara et al., 2015) as well as to increase expression of OT (hypothalamus) (Yuan et al., 2020) and OTR (BAT and IWAT) (Yuan et al., 2020) and levels of circulating OT (Yuan et al., 2020). One study by Kasahara et al. (2013) reported no change of PVN or SON OT mRNA expression in response to cold but the data were not shown in the study. Mice that lack OT or its receptor do have notable impairments in cold-induced thermogenesis (Kasahara et al., 2007, 2013, 2015; Takayanagi et al., 2008; Xi et al., 2017) and enlarged lipid droplets in IBAT (suggestive of hypo-activity) (Takayanagi et al., 2008) which would potentially contribute to impairments in energy expenditure. Deficits in PVN OT signaling or pharmacological blockage of OT receptors are associated with defects in energy expenditure (Zhang and Cai, 2011; Zhang et al., 2011; Wu et al., 2012). It is well appreciated that BAT thermogenesis is important in the regulation of energy expenditure (see Cannon and Nedergaard, 2004; Morrison et al., 2014, for review), but it is not clear if OT’s effects on energy expenditure result from BAT thermogenesis. One recent report measured both energy expenditure and BAT thermogenesis in mice with diphtheria toxin-elicited reductions of PVN and SON OT signaling and found reductions in both IBAT temperature and core temperature in response to a cold stimulus, both of which were attenuated by OT pre-treatment (1 mg/kg, IP). However, there were no alterations in IBAT temperature, core temperature or energy expenditure in mice that were housed at 20–24°C (Xi et al., 2017). Similar to the findings by Xi et al. (2017), Kasahara et al. (2013) found a reduction in core temperature in OTR deficient mice that were exposed to cold and also no change in energy expenditure between genotypes at room temperature. Whether their findings point to a more important role of endogenous OT in the control of cold-induced thermogenesis and cold-induced elevations of energy expenditure will remain to be determined. It will be important to determine in future studies if mice with global loss of OT or OT receptors have impairments in both BAT thermogenesis and energy expenditure in response to cold exposure and whether pre-treatment with OT rescues both the impairments in BAT thermogenesis and energy expenditure. This could shed light on whether impairments in BAT thermogenesis may also be linked to impairments in energy expenditure in these animals.

### What Receptor Populations Mediate Oxytocin’s Effects on Brown Adipose Tissue Thermogenesis and Energy Expenditure?

Paraventricular nucleus OT neurons are anatomically positioned to control BAT thermogenesis and energy expenditure through polysynaptic projections to IBAT (Oldfield et al., 2002), stellate ganglia (Jansen et al., 1995) [sympathetic ganglia known to innervate IBAT (Oldfield et al., 2002)], as well as WAT depots [EWAT (Shi and Bartness, 2001; Stanley et al., 2010) and IWAT depots (Shi and Bartness, 2001)]. Oldfield et al. (2002) determined that OT was expressed in approximately 10–15% of PVN neurons that were also co-infected with pseudorabies virus following injections into IBAT. This is in contrast to vasopressin, cocaine and amphetamine-regulated transcript and corticotropin-releasing factor, which were rarely found in PVN OT neurons that were labeled with pseudorabies virus (PRV). Similarly, Jansen et al. (1995) reported that OT was expressed in 10% of PVN neurons that were co-infected with PRV following injections into stellate ganglia and was also found to be more commonly expressed in PRV (+) neurons than vasopressin (2%), CRH (5%) and thyrotropin-releasing hormone (<1%). Shi and Bartness found that 3.49% of PVN OT neurons were co-labeled with PRV following PRV injections into WAT (Shi and Bartness, 2001), higher than vasopressin (1.07%) and tyrosine hydroxylase (2.62%). In a separate study, Stanley et al. (2010) administered PRV into EWAT and determined that up to 17% of neurons that expressed PRV also expressed OT compared to approximately 12, 4 and 26% for vasopressin, TRH and CRH. Collectively, these findings suggest that OT is one of the more predominant peptides found in outgoing projections to IBAT, IWAT and EWAT.

The extent to which specific OTR populations contribute to BAT thermogenesis and energy expenditure have been examined by determining (1) the effects of localized administration of OT on BAT or core temperature, (2) the activation of regions that express OTRs in response to cold and (3) whether deficits in cold-induced thermogenesis in OTR deficient mice can be restored by re-expression of OTRs into specific CNS sites. Central (3V) administration of OT (which does not differentiate forebrain receptor populations or a forebrain vs. hindbrain site of action) has been found to increase BAT temperature in both mice and rats (Roberts et al., 2017). Noble et al. (2014) extended these findings by showing that OT administration into the hypothalamus (VMH) stimulated energy expenditure in rats, thereby providing more direct evidence in support of a role of VMH OTRs in the control of energy expenditure. In addition, Kasahara showed that cold exposure activates both PVN OXT neurons (Kasahara et al., 2007) and neurons within the dorsomedial nucleus (DMN), an area that expresses OTRs) (Kasahara et al., 2013). Kasahara et al. (2013) also noted that cold also stimulated number of c-Fos(+) neurons (marker of neuronal activation) and that this effect was attenuated in OTR deficient mice. Kasahara et al. (2013) further determined the extent to which OTR signaling within the DMH/VMH was sufficient to elicit BAT thermogenesis by measuring BAT thermogenesis in OTR null mice that received adeno-associated viral vector expression of OTRs in DMH/VMH (Kasahara et al., 2013). They found that OTR expression within the DMH/VMH restored deficits in cold-induced thermogenesis and corrected defects in β3- and α2-adrenoceptor mRNA expression in IBAT. Together, these findings indicate that OTR signaling within the DMH and/or VMH is sufficient to elicit BAT thermogenesis.

The role of OTRs within the midbrain raphe nucleus (median raphe) in the control of body temperature has also been explored. Yoshida et al. (2009) found that direct injections of OT into the median raphe increase body temperature supporting a role of OTRs in this region in the regulation of BAT thermogenesis.

In addition to the midbrain raphe and hypothalamic OTRs, several studies have suggested that hindbrain OTRs may also be important in contributing to OT-elicited BAT thermogenesis. Namely, 4V administration (to target hindbrain OTRs) of OT increases BAT temperature in both rats (Roberts et al., 2017) and mice (Edwards et al., 2021b). In addition, Ong et al. (2017) found that 4V administration also increases core temperature in a rat model. Kasahara extended these findings and probed the role of hindbrain OTRs within the rostral raphe pallidus, a region that receives dense innervation from the PVN (Luo et al., 2019), is a component of rostral medullary raphe (RMR) and contains premotor neurons with polysynaptic projections to BAT (Oldfield et al., 2002) and to stellate ganglia that innervate BAT (Jansen et al., 1995). They found that OTR (+) neurons within the rostral raphe pallidus are activated in response to cold exposure (Kasahara et al., 2015). They subsequently addressed if OTRs within the RMR were sufficient to elicit BAT thermogenesis and found that expression of OTRs within the RMR restored deficits in cold-induced thermogenesis and reduced the size of the lipid droplets in IBAT tissue to that of control mice (Kasahara et al., 2015). Kasahara et al. (2015) also addressed if OTR in the RMR were necessary for thermoregulation by using an AAV-Cre to delete OTR expression from the RMR of Oxtr^fx/fx^ mice. However, AAV-Cre- elicited deletion of OTRs within the RMR was not effective in restoring impairments in core temperature in response to cold stimulus. The NTS is another hindbrain site that expresses OTRs and has outgoing polysynaptic projections to IBAT (Oldfield et al., 2002) and to stellate ganglia that innervate BAT (Jansen et al., 1995). It is not clear if OTR (+) neurons project to rostral raphe pallidus or directly to the spinal cord. Future studies assessing the impact of NTS OTR gain of function and loss of function will help further delineate the role of NTS OTRs in the control of BAT thermogenesis. Together, these findings suggest that OTRs within multiple hindbrain areas may contribute to the effects of OT on BAT thermogenesis.

In addition to acting at OTRs within the brain, OT may also stimulate BAT thermogenesis and energy expenditure through sympathetic pre-ganglionic OTR-expressing neurons within the spinal cord. Chemogenetic stimulation of OT neurons within the rostral PVN, some of which innervate the thoracic spinal cord, increases c-Fos in thoracic spinal cord cholinergic neurons, boosts energy expenditure and tends to elevate IBAT temperature (*P* = 0.13) (Sutton et al., 2014), although the extent to which these effects are attributed to endogenous OT or another peptide/neurotransmitter within OT neurons has not been determined. Finally, a recent study indicated that OT may also have a direct action through OT on OTRs on brown adipocytes within BAT (Yuan et al., 2020) where OTR expression is found to be upregulated in response to cold (Yuan et al., 2020). Future studies should address if targeted disruption of OTRs within the forebrain hypothalamus, midbrain, hindbrain, spinal cord and BAT decrease both cold-induced thermogenesis and elevations of energy expenditure and elicit adult-onset obesity (similar to that of global OT or OTR deficient mice).

## Does Locomotor Activity Contribute to the Ability of Oxytocin to Increase Interscapular Brown Adipose Tissue Temperature and Energy Expenditure and Evoke Weight Loss?

In addition to BAT thermogenesis and heat production, increased locomotor activity is another mechanism to stimulate energy expenditure. However, current data suggest that OT’s effects on locomotor activity are inconsistent and appear to vary depending on how it was administered (chronic vs. acute) and, in some cases, whether the animals were lean or obese. We and others have also found that chronic infusions of OT into the lateral ventricle or 3V, at a dose that was sufficient to reduce body weight and elevate IBAT temperature (16 nmol/day), had no effect on locomotor activity in DIO rats (Deblon et al., 2011; Blevins et al., 2015; Roberts et al., 2017). In addition, Maejima et al. (2011) have also found that subcutaneous infusion of OT, at a dose that was sufficient to reduce body weight, also had no effect on locomotor activity in DIO mice (1.6 mg/kg/day or ∼56.4 nmol/day). Based on these collective findings, OT-elicited increases in locomotor activity do not appear to be a major contributor to OT-elicited weight loss in DIO models.

Chemogenetic stimulation of PVN OT neurons or acute CNS or systemic administration of OT has been found to either stimulate, reduce or have no effect on locomotor activity. Sutton et al. (2014) found that chemogenetic activation of PVN OT neurons in *Oxytocin-ires-Cre* mice increased locomotor activity and energy expenditure and resulted in a tendency (non-significant) toward an increase in subcutaneous IBAT temperature in animals with transponders implanted above the IBAT depot. A recent study by Zhang et al. (2021) found that chemogenetic activation of magnocellular OT neurons that project to the striatum, results in stimulation of locomotor activity over a 20-min period though an OTR-dependent mechanism. Similar to the forementioned chemogenetic studies, one study found that acute administration of OT into the VMH stimulated physical activity in rats at 1-h post-injection (Noble et al., 2014), but these effects were short-lived and did not following the more prolonged effects of 3V or 4V OT on IBAT temperature in rats (Roberts et al., 2017) and mice (Roberts et al., 2017; Edwards et al., 2021b). Additional findings indicate that systemic OT was found to (1) reduce methamphetamine-elicited elevations in locomotor activity in rats (Carson et al., 2010), (2) reduce locomotor activity in rats in an OTR-dependent manner (Angioni et al., 2016), or (3) have no significant effect on locomotor activity in female ovariectomized (Iwasa et al., 2019b) or perimenopausal rats (Erdenebayar et al., 2020). Consistent with the earlier reports that systemic OT was able to reduce locomotor activity, CNS administration of OT was also found to block the effects of CNS administration of OTR antagonist to increase locomotor activity. Maejima et al. (2015) also found that intranasal OT, at a dose that reduces food intake, had no effect on locomotor activity while systemic (IP) administration reduced locomotor activity but only during the dark cycle (Maejima et al., 2015). Collectively, these studies suggest that OT results in a brief elevation of locomotor activity, has no effect or reduces locomotor activity. In order to assess if locomotor activity may play a role in OT-elicited elevations of IBAT temperature and/or energy expenditure, IBAT temperature, energy expenditure and locomotor activity should be measured in parallel in the same animals under the same conditions.

## Is Oxytocin Effective at Reducing Body Weight in Male and Female Rodents?

Much of the early historical work regarding the effects of OT in the control of food intake has focused on male rodent models but the few studies that have been completed in female rodents have produced somewhat mixed results. One early study reported that acute central and peripheral administration of OT reduced food intake similarly in both male and female rats (Benelli et al., 1991). Subsequently, Maejima et al. (2017) examined the effects of OT on body weight and adiposity and found that chronic sc infusions of OT produced similar reductions on body weight and adiposity in male and female DIO C57Bl/6J mice. In addition, central [intracerebroventricular (ICV)] administration of OT was found effective at reducing food intake and weight gain in female genetically obese Sim1 haploinsufficient mice but not in female wild-type counterparts (Kublaoui et al., 2008). A recent study by Seelke et al. (2018) reported that intranasal OT may have a more heightened response to reduce weight gain in female DIO prairie voles but the sample size was small. Iwasa et al. (2019b) reported that chronic systemic OT (1x IP administration over 6 days) treatment reduced food intake and body weight in female ovariectomized rats. One recent study by Liu et al. (2020a) indicate that the effectiveness of central OT (ICV) administration to reduce food intake in female rats is influenced by estrous cycle (particularly proestrous) and that estrogen replacement in ovariectomized rats inhibits OT’s effects on food intake. In light of these recent findings and the earlier work by Maejima in DIO mice, it will be important to determine if other routes of administration are as impacted by estrous cycle and whether the ability of chronic central or systemic administration of OT to elicit weight loss in female rodents can be optimized if given intermittently throughout the estrous cycle.

The role of endogenous OT in the control of body weight in female rodent models is not clearly understood due, in part, to inconsistent results and data that has largely been generated in male rodents. Previous studies indicate that male OT null mice develop adult-onset obesity at 10 (Kasahara et al., 2007) or 16 weeks (Camerino, 2009) while OTR receptor null mice develop adult-onset obesity at 12 weeks (Takayanagi et al., 2008). While female OT null mice also develop adult onset obesity (Camerino, 2009; Tamma et al., 2009) as early as 8 weeks (Tamma et al., 2009), female OTR deficient mice fail to develop increased body weight relative to control counterparts (Takayanagi et al., 2008; Sun et al., 2019). However, both male and female OT [3–10 months: females; 8–11 months: males] and OTR null mice [3 months: males and females] develop increased fat mass and/or percent fat (Sun et al., 2019). In contrast to the findings from male and female OT null mice, only male *Oxytocin-IresCre:Rosa26^iDTR/+^* mice with diphtheria toxin-elicited ablations of PVN OT neurons become obese relative to control *Rosa26*^iDTR/+^ mice (Wu et al., 2012). In contrast to the study from Takayanagi et al. (2008), male OTR^–/–^ mice failed to show increased body weight relative to wild-type controls (Sun et al., 2019) (personal communication with Dr. Tony Yuen and Dr. Mone Zaidi) although it is certainly possible that they would have become obese over time. Further studies will be required in order to provide more clarity on whether differences in genetic background, housing, thermoregulation and/or age might be contributing factors in terms of these apparent differences across studies.

## How Does Oxytocin Impact Body Composition?

Oxytocin may impact body composition through a direct effect on OTRs on adipocytes which express OTRs (Muchmore et al., 1981; Schaffler et al., 2005; Tsuda et al., 2006; Altirriba et al., 2014; Gajdosechova et al., 2014, 2015; Yi et al., 2015) or through an indirect effect through outgoing polysynaptic projections from the PVN to both IWAT (Shi and Bartness, 2001) and EWAT (Shi and Bartness, 2001; Stanley et al., 2010).

Chronic administration (repeated injections or minipump infusions) into the CNS (lateral ventricle, 3V) or systemic OT treatment was found to decrease fat mass relative to baseline fat mass (pre-intervention) or decrease fat mass post-treatment in lean (Deblon et al., 2011) and DIO rats (Sprague-Dawley CD^®^ IGS, Long-Evans and Wistar rats) (Deblon et al., 2011; Morton et al., 2012; Blevins et al., 2016; Roberts et al., 2017), DIO C57BL/6J (Roberts et al., 2017) and C57BL/6 mice (Zhang and Cai, 2011; Snider et al., 2019), *db/db* mice (Plante et al., 2015), and *ob/ob* mice (Altirriba et al., 2014) without producing any significant reductions in lean mass. OT (repeated IP administration) was also found to reduce percent fat mass after only 1 week of treatment and these effects were not associated with any effects on lean mass in lean wild-type mice (Sun et al., 2019) (1 μg/mouse, IP, 3x/week). These more selective effects on fat mass in the absence of any adverse effects on lean mass have also been recapitulated following chronic hindbrain (4V) infusions in DIO C57BL/6J mice (Edwards et al., 2021b).

Oxytocin treatment appears to reduce sc (Maejima et al., 2017), mesenteric (Maejima et al., 2011), EWAT (Maejima et al., 2011; Altirriba et al., 2014) and visceral fat (Maejima et al., 2017) in DIO C57Bl/6J (Maejima et al., 2011) and *ob/ob* mice (Altirriba et al., 2014) as well as in female ovariectomized Wistar rats (Iwasa et al., 2019b). In addition, OT reduced adipocyte size across several fat depots including sc (Plante et al., 2015; Iwasa et al., 2019b)/inguinal (Edwards et al., 2021b), visceral (Iwasa et al., 2019b), perirenal (Plante et al., 2015), and epicardial (Plante et al., 2015), EWAT (Eckertova et al., 2011; Maejima et al., 2011; Balazova et al., 2016) and mesenteric (Maejima et al., 2011) depots in C57BL/6J (Maejima et al., 2011) and *db/db* mice (Plante et al., 2015) as well as obese Zucker rats (Balazova et al., 2016) or female ovariectomized Wistar rats (Iwasa et al., 2019b). Chronic sc OT treatment also reduced liver weight and fat in hepatocytes in DIO C57BL/6J mice (Maejima et al., 2011) but was found to have no effect on liver triglyceride content in *ob/ob* mice (Altirriba et al., 2014). In contrast, some studies have reported that chronic systemic or central (4V) OT administration elicited a relative reduction in lean mass compared to vehicle in lean C57BL/6J (Altirriba et al., 2014) and DIO C57BL/6 mice (Snider et al., 2019) as well as in DIO CD IGS rats (Roberts et al., 2017) with or without any relative reductions in fat mass raising the possibility that differential effects may be attributed, in part, to rodent strain, dosing and/or route of administration. While there are exceptions, overall OT treatment appears to preferentially reduce fat mass while preserving lean mass across rodent models.

More recent studies have found that more translational routes of administration (intranasal) have also yielded promising effects on body composition in rodents and humans. Chronic intranasal administration (8 IU/kg; 1x daily over 7 days) tended to reduce carcass fat mass in DIO prairie voles without negatively impacting lean mass (Seelke et al., 2018). Recent translational studies in humans indicate that chronic intranasal OT (24 IU; 4x daily for 8 weeks) administration also tended to reduce fat mass in obese men and women (Espinoza et al., 2021). Interestingly, it also produced a slight increase in lean mass (Espinoza et al., 2021). These findings are in agreement with previous studies that found both a reduction in relative fat mass (pre- vs. post-intervention) and a slight increase in relative lean mass (pre- vs. post-intervention) following 3V administration (16 nmol/day; ∼3-week body comp measurements) (Blevins et al., 2016) and subcutaneous administration (50 nmol/day) in chow-fed and DIO rats (10-day body comp measurements). Whether these effects on lean mass are due, in part, to OT’s effects on muscle mass and/or bone composition merit further investigation and is discussed further in Section “Mechanism of Action Following Peripheral Administration.”

Existing data suggest that OT may impact body composition through increased lipolysis or reduced lipogenesis. *In vitro* data indicate that OT stimulates glycerol and free fatty acids and/or reduces triglycerides in 3T3-L1 adipocytes (Deblon et al., 2011; Yi et al., 2015). These findings have been recapitulated *in vivo* in rats (Deblon et al., 2011) and DIO non-human primates (rhesus monkeys) (Blevins et al., 2015). Chronic intranasal OT tended to reduce triglycerides in pre-diabetic obese humans but these effects were not reach statistical significance (Zhang et al., 2013). In addition, chronic ICV infusions of OT (1.6 and 16 nmol/day) increases expression of hormone sensitive lipase (an enzyme linked with lipolysis) in DIO rats (Deblon et al., 2011) in EWAT. In addition, these effects on EWAT were reproduced following chronic sc infusions of OT (50 nmol/day) in *ob/ob* mice (Altirriba et al., 2014) as well as direct application of OT to 3T3-L1 adipocytes (5 μm OT over 24 h) (Deblon et al., 2011). These findings implicate that these pro-lipolytic effects may occur through both a direct and indirect mechanism. OT was also found to decrease expression of fatty acid synthase (an enzyme linked to lipogenesis) in *ob/ob* mice (Altirriba et al., 2014), indicating that OT may also decrease lipogenesis. Existing data from animal models suggest that chronic central or systemic OT infusions reduce respiratory quotient in DIO rats (Deblon et al., 2011) and mice (Maejima et al., 2011) compared to vehicle treatment (Deblon et al., 2011; Maejima et al., 2011) or pair-fed animals (Deblon et al., 2011). These effects were also recently translated to humans as OT was also found to reduce respiratory quotient in lean and obese men (Lawson et al., 2015). Collectively, these findings suggest that OT reduces body adiposity and adipocyte size by increasing lipolysis and lipid utilization or oxidation and reducing lipogenesis.

## How Does Oxytocin Impact Muscle Mass and Bone Composition?

### Effects of Oxytocin on Muscle Mass

Recent studies implicate a role for OT in the control of thermogenesis in skeletal muscle and muscle regeneration. OTR are expressed in skeletal muscle (Elabd et al., 2014; Gajdosechova et al., 2014, 2015) and in C2C12 mouse myoblast cells (Lee et al., 2008). Existing data suggest that OT may elicit direct effects on OTRs in skeletal muscles as (1) OT was found to stimulate intracellular calcium in C2C12 mouse myoblast cells and (2) chronic sc infusions of OT stimulate markers of thermogenesis in skeletal muscle (*UCP-3* and *Atb5a1*) (Yuan et al., 2020). In addition to recently recognized role of skeletal muscle thermogenesis, OT has been found to have an important role in muscle regeneration. Loss of function studies show that OT deficient mice have impairments in muscle regeneration @ 12 months of age, but such impairments were not observed in younger animals (3 months of age) (Elabd et al., 2014). These data are consistent with the finding that OTR deficient mice at 3 months of age do not have any impairments in lean mass relative to age-matched wild-type mice (Sun et al., 2019). In addition, hind limb muscles (gastrocnemius and tibialis anterior) in OT deficient mice are associated with reduced muscle mass. The hind limb muscles (quadriceps, gastrocnemius, and tibialis anterior) in OT deficient mice were found to be associated with increased perimuscular and intermuscular adipose tissue (Elabd et al., 2014), findings which are consistent with increased body weight and/or fat mass in this mouse model (Kasahara et al., 2007; Camerino, 2009; Sun et al., 2019).

Of translational importance is that finding that chronic intranasal OT was found to increase lean mass in senior men and women with sarcopenic obesity (discussed in section “Is Oxytocin Effective at Reducing Body Weight in Male and Female Rodents?”). This is consistent with the finding that positive associations have been observed between overnight serum OT concentrations and lean mass in premenopausal women (Schorr et al., 2017). A separate study using animal models provided potential mechanistic insights into these effects. Systemic (sc) treatment with OT (1 μg/g) was able to improve muscle regeneration in aged mice to a level that as comparable to that of younger mice (Elabd et al., 2014). In these studies, OT was found to increase (1) the proliferative capacity of old satellite myogenic cells to that of young satellite myogenic cells and (2) the proliferation of primary myogenic progenitor cells. The effects of OT on satellite cells were also found to occur through the MAPK/ERK signaling pathway. Collectively, these findings provide supportive data for future mechanistic studies using intranasal OT in humans with sarcopenia.

### Effects of Oxytocin on Bone Composition

In animal models, OT has been found to have a critical role in maintaining bone mass in both male and female mice as both OT and OTR null mice develop osteoporosis (Tamma et al., 2009) (see Colaianni et al., 2015; McCormack et al., 2020, for review). The effects of OT on bone formation are likely due to a direct action of circulating OT as peripheral OTRs as OTRs are expressed on both mouse (Tamma et al., 2009; Colaianni et al., 2012) and human osteoblasts (Copland et al., 1999; Tamma et al., 2009) and osteoclasts (Colucci et al., 2002; Tamma et al., 2009). Furthermore, peripheral administration of OT (two IP injections separated by 12 h; 4 μg/mouse) increased TRAP-positive osteoclast formation while central administration was ineffective (Tamma et al., 2009). Peripheral administration has also been found to increase bone mineral density and osteoblast formation, proliferation and differentiation (Tamma et al., 2009). Sun et al. (2019) recently determined that OTRs on osteoblasts were critically important in bone formation by ablating OTRs in osteoblasts using Col2.3Cre mice. Male and female mice that lack OTRs in osteoblasts were found to have low bone mass that resembled that of the OTR null mice (Sun et al., 2019). Mice that lacked OTRs in osteoclasts also developed high bone mass in Acp5Cre mice suggests an important role of OT to increase osteoclastogenesis. Collectively, the effects of OT on bone are thought to occur through both osteoblast differentiation as well as regulation of osteoclast development and function.

While less is known from a mechanistic standpoint about OT-elicited regulation of bone mass in humans, human studies do reveal that there is a positive association between bone mass and levels of circulating OT in women (Schorr et al., 2017). While Breuil et al. (2015) did not find this positive association in men, it did find a weak negative association between circulating OT and fracture risk. Breuil et al. (2011, 2014) also found that circulating OT levels correlated with osteopenia or osteoporosis in post-menopausal women. In particular, higher circulating levels of OT were associated with high bone mineral density (hip) in women with lower circulating levels of estradiol or higher circulating levels of leptin (Breuil et al., 2014). Similar to what is observed in OT and OTR null mice (Tamma et al., 2009) and in Col2.3Cre mice that lack OTRs in osteoblasts (Sun et al., 2019), humans with low OT serum levels display severe osteoporosis (Breuil et al., 2011). Overall, the effects of OT on bone mineral density in humans appears to be complicated and influenced by sex steroids and metabolic status.

## Mechanism of Action Following Peripheral Administration

While systemic administration of OT may impact body adiposity through a direct effect on white and brown adipocytes, it may reduce food intake, in part, through a direct action on peripheral OTRs in the GI tract (Wu et al., 2003, 2008; Monstein et al., 2004; Ohlsson et al., 2006; Qin et al., 2009; Welch et al., 2009), the enteric nervous system (Monstein et al., 2004; Welch et al., 2009), smooth muscle cells (Monstein et al., 2004; Qin et al., 2009) and the vagus nerve (Welch et al., 2009; Brierley et al., 2021) as well as central OTRs (Ring et al., 2006, 2010; Ho et al., 2014; Iwasaki et al., 2019). Peripheral administration of OTR antagonists that are capable of crossing the BBB stimulate food intake in rodents (Olszewski et al., 2010; Zhang and Cai, 2011) which implicate OTRs within either the CNS or periphery in the control of food intake. Subsequent studies showed that peripheral administration of an OTR antagonist that is not thought to readily cross the BBB (L-371,257) produced modest effects to stimulate food intake and body weight gain in chow-fed rats (Ho et al., 2014) suggesting the potential importance of peripheral OTRs in the control of energy balance. Iwasaki et al. (2014, 2019) extended these findings in two separate studies and found that the ability of peripheral administration of OT to reduce food intake and elicit Fos (marker of neuronal activation) in the PVN and hindbrain was either attenuated (0.4 mg/kg, IP) or blocked (0.2 and 0.4 mg/kg, IP) in capsaicin-treated and vagotomized mice. Collectively, these findings suggest that OTR signaling through vagal afferents contributes to the effects of peripheral OT administration to reduce food intake. Furthermore, a recent study found that NTS preproglucagon neurons are critical downstream meditators of the feeding suppression in response to systemic OT (0.4 mg/kg, IP) (Brierley et al., 2021). Together, these findings are consistent with a role of peripheral OTRs in contributing to the effects of systemic OT.

One potential mechanism by which peripheral OT may reduce food intake is through the reduction of gastric emptying. Peripheral OT treatment also decreases gastric emptying in rodents [mice (Welch et al., 2014)/rats (Wu et al., 2003, 2008)] and these effects are attenuated following treatment with an OTR antagonist, Atosiban (Wu et al., 2003, 2008), indicating that these effects are attributed to OTRs. In other cases, systemic administration has been found to have effect on gastric emptying rate [rats (McCann et al., 1989)/humans (Borg et al., 2011)] or a stimulatory effect on gastric motility [rabbits (Li et al., 2007)]. Whether these effects can be attributed, in part, to dosing or species differences awaits further investigation.

Whether the peripheral effects of OT on gastric emptying in rodents is mediated, in part, through activation of peripheral or central OTRs is still not known. CNS administration of OT reduces gastric motility (Rogers and Hermann, 1987; Flanagan et al., 1992) and these effects appear to be mediated by OTRs in the dorsal vagal complex (Rogers and Hermann, 1987). While circulating OT may inhibit gastric motility through a central mechanism these effects may also be mediated through stimulation of OTRs that are expressed in the enteric nervous system (Monstein et al., 2004; Welch et al., 2009) on smooth muscle cells (Monstein et al., 2004; Qin et al., 2009) or nodose ganglion (Welch et al., 2009). As mentioned earlier, systemic OT suppresses food intake, in part, through a vagal mechanism (Iwasaki et al., 2015, 2019) and signaling through NTS preproglucagon (PPG) neurons (Brierley et al., 2021). Future studies that address if the ability of systemic OT to decrease gastric emptying is impaired in capsaicin-treated or vagotomized rodents will help to differentiate a central from peripheral mechanism of action.

Oxytocin may also reduce gastric emptying through the local release of cholecystokinin-8 (CCK-8) and subsequent activation of vagal afferents that innervate the hindbrain. Systemic administration of OT inhibits both gastric emptying and stimulates the release of CCK-8 (Wu et al., 2003, 2008), both of which occur within the time period that peripheral administration of OT reduces food intake (Ho et al., 2014). In addition, the effects of systemic OT to reduce gastric emptying are blocked by pretreatment with a CCK1 receptor antagonist, devazepide (Wu et al., 2003, 2008). Furthermore, Iwasaki showed that peripheral administration of OT and CCK-8 both activate single vagal afferent neurons (Iwasaki et al., 2014) further supporting both a direct and indirect action of OT to activate vagal relays through activation of CCK1 receptors (Wu et al., 2008). Further studies to determine if the effectiveness of systemic OT to suppress food intake and reduce gastric emptying is attenuated in animals with gastric fistulas that are open (sham feeding; no gastric distension) relative to animals with closed fistulas (real feeding; gastric distension) will be helpful in determining if OT inhibits food intake, in part, by suppressing gastric emptying.

The extent to which circulating OT may inhibit food intake through suppression of the orexigenic signal, ghrelin, is controversial. It has been reported that ghrelin administration centrally can stimulate OT release in rodents (Szabo et al., 2019) and heterocomplex formed by OT receptor and ghrelin receptor can alter OT signaling (Wallace Fitzsimons et al., 2019) (see section “Mechanism of Action Following Peripheral Administration”). One study reported that peripheral OT treatment decreased circulating levels of ghrelin in men (Vila et al., 2009) during a time that is consistent with when OT reduces food intake in rodents (Ho et al., 2014). In contrast, intranasal administration of OT, at a dose that reduced total caloric intake (Lawson et al., 2015), cookie consumption (Ott et al., 2013) and increased circulating levels of OT in other studies (Burri et al., 2008; Striepens et al., 2013), failed to reduce plasma ghrelin. It will be helpful to examine the impact of chronic intranasal OT on circulating levels of ghrelin in the setting of weight loss as this could offer potential mechanistic insights into downstream targets of OT action and additional insights into how OT reduces energy intake in humans, perhaps impacting both homeostatic and reward-based food intake in humans.

## Effects of Intranasal Oxytocin on Energy Homeostasis in Rodent Models

The extent to which circulating OT may enter the CNS remains controversial (Mens et al., 1983; Ermisch et al., 1985; Kendrick et al., 1986; Ring et al., 2006, 2010; Neumann et al., 2013; Ho et al., 2014). Circulating OT may have limited or restricted access to the CNS although some studies suggest that OT does cross the BBB (Mens et al., 1983; Ermisch et al., 1985), and CNS sites that are leaky to BBB (e.g., median eminence and area postrema) might serve as sites of OT uptake. It is also unclear whether transport of OT across the BBB could be hampered in the DIO state as has been proposed for other hormones such as leptin (Banks et al., 1999). Leng and Sabatier (2017) have also raised the possibility that with high peripheral doses, “some OT is likely to enter the brain despite the presence of a very effective blood–brain barrier to OT.” Consistent with this, Freeman et al. (2016) found elevated levels of OT within the CSF following the highest intravenous dose of OT used in their study (5 IU/kg or ∼29–36.5 IU). Lee et al. (2018) also found that deuterated OT given intravenously at a higher dose (80 IU) also resulted in elevated levels within the CSF of rhesus monkeys. In addition, others found that central administration of a non-penetrant OTR antagonist, L-371,257, was able to block the anxiolytic effects of peripheral administration of OT (Ring et al., 2006). In addition, we have also generated data to suggest that hindbrain OTRs contribute, in part, to the satiety response to peripherally administered OT (Ho et al., 2014). In an effort to target the CNS more directly using a minimally invasive route of administration, the majority of clinical trials have administered OT by the intranasal route of administration.

Intranasal administration enables relatively rapid uptake into the CSF of several neuropeptides and hormones, including insulin, vasopressin, and the melanocyte-stimulating hormone, adrenocorticotrophic hormone (4–10), within 30 min in humans (Born et al., 2002). Intranasal delivery into the cribriform plate rather than the turbinates is one approach that has been proposed to maximize delivery to the CNS and limit uptake into the circulation (Meredith et al., 2015). Intranasal delivery appears to effectively enable OT to enter the CSF in mice, rats, non-human primates and humans within 30–45 min post-treatment (Gossen et al., 2012; Neumann et al., 2013; Chang and Platt, 2014; Monte et al., 2014) although others have found that only aerosolized OT (24 IU) reached the CSF of non-human primates (Modi et al., 2014) while intranasal OT @ 24 IU and IV OT @ 48 IU was ineffective. In addition, the extent to which intranasal OT may reach the parenchyma from the CSF is being debated (see Leng and Ludwig, 2016a,b; Leng and Ludwig, 2016c, for review). As Leng and Ludwig (2016a) stated: “several recent studies have looked at the effects of intranasal application of OT on food intake in man. These involve very high doses of OT that raise plasma concentrations to supraphysiological levels; a small amount of the applied OT probably reaches the brain, but whether it does so in effective amounts is uncertain.” In addition, the degree to which the OT measured in CSF in response to intranasal OT is due to elevations of exogenous or endogenous OT is also controversial (Ludwig et al., 2013; Neumann et al., 2013; Lee et al., 2018; Smith et al., 2019). Similar to vasopressin (Gouzenes et al., 1998), OT is one of the few hormones that is can stimulate its own release. This can occur, in part, through magnocellular SON (Yamashita et al., 1987; Moos and Richard, 1989) and PVN (Kendrick, 2000) OT auto-receptors following either central or systemic administration. Systemic OT can do this indirectly by activating vagal afferents (Kendrick, 2000; Iwasaki et al., 2015, 2019) where OTRs are expressed (Welch et al., 2009), increasing Fos within PVN OT neurons (Carson et al., 2010; Hicks et al., 2012; Hayashi et al., 2020) and stimulating release of OT within the CNS (Zhang and Cai, 2011) and likely back into the peripheral circulation. These findings have been recently extended to high fat diet-fed mice where systemic OT treatment was recently shown to increase Fos within PVN OT neurons (Hayashi et al., 2020) and up-regulate hypothalamic OT mRNA. Similarly, chronic CNS infusions of OT can up-regulate hypothalamic OT mRNA and increase OT levels within the circulation (Miller, 2013). The finding that circulating levels of OT are elevated at 15 (Striepens et al., 2013), 30 (Striepens et al., 2013), 40 (Monte et al., 2014), 45 (Striepens et al., 2013), 60 min (Striepens et al., 2013; Kirkpatrick et al., 2014; Lee et al., 2018), or 90 min (Kirkpatrick et al., 2014) following intranasal administration in non-human primates (Monte et al., 2014; Lee et al., 2018) and humans (Striepens et al., 2013; Kirkpatrick et al., 2014) raise the possibility that intranasal OT may be either entering the circulation directly or indirectly following the release of endogenous OT into the CNS and peripheral circulation. To address the possibility that the rise in CNS OT in response to intranasal OT might be a result of endogenous release, Smith, Korgan and Young examined the extent to which intranasal OT entered the CNS in OT null mice (Smith et al., 2019). They found that intranasal OT elevated OT levels within the left amygdala as well as blood (Smith et al., 2019). Collectively, these findings extend the previous findings in rodents, non-human primates and humans and suggest that OT is capable of entering the parenchyma following intranasal delivery.

One question is how translatable are metabolic data that are generated in animal models following central or peripheral administration given that OT is largely being administered intranasally in humans. As previously discussed, OT given by peripheral route of administration is likely to act by both peripheral and central OTRs (particularly at higher doses) to inhibit food intake and potentially stimulate thermogenesis and energy expenditure. There is a very limited amount of metabolic data following intranasal administration in rodent models, but it has been found to largely recapitulate the effects of central and peripheral OT to reduce food intake (Maejima et al., 2015) and/or weight gain (Seelke et al., 2018) in mice and DIO prairie voles, respectively. While additional studies tracking the effects of chronic intranasal administration on food intake, thermogenesis and energy expenditure and weight loss need to be undertaken in the rodent model, the data obtained from both central and peripheral administration in animal models appears to translate well to current findings following intranasal administration in animal models.

## Effects of Oxytocin on Dyslipidemia and Lipolysis in Rodent and Non-Human Primate Models

Both *in vitro* and *in vivo* data suggest that OT reduces dyslipidemia and increases lipolysis in rodent and non-human primate models. OT was found to stimulate glycerol in 3T3-L1 adipocytes (Yi et al., 2015), which express OTRs (Schaffler et al., 2005; Yi et al., 2015), indicating that OT stimulates lipolysis in this model through a direct action. In addition, OT also stimulated glycerol release from epididymal fat pads *ex vivo* (Deblon et al., 2011). ICV OT (1.6 nmol/day) also increased serum glycerol and reduced serum triglycerides following 2-week treatment in rats (Deblon et al., 2011). Chronic 3V infusions (16 nmol/day) over 21–28 days was found to reduce total cholesterol in DIO mice (Roberts et al., 2017) and rats (Blevins et al., 2016; Roberts et al., 2017). Recent findings indicate that in female perimenopausal rats, systemic OT over 12 days was found to reduce triglycerides, LDL and HDL cholesterol (Erdenebayar et al., 2020) raising the possibility that OT could be beneficial in treating hyperlipidemia at the time of menopause or post-menopause. Blevins extended these findings in DIO non-human primates (rhesus monkeys) where chronic 2 × daily subcutaneous injections of OT reduced total cholesterol, Apolipoprotein C-III, high-density lipoprotein, serum triglycerides and increased serum free fatty acids and glycerol following 4-weeks of treatment. It was also associated with a transient reduction of low-density lipoprotein (Blevins et al., 2015). One additional study found that chronic ICV (1.6 nmol/day) infusions of OT stimulated EWAT mRNA expression of lipoprotein lipase (Lpl) and fatty acid transporter (fat) (Deblon et al., 2011), which have both been linked uptake of triglycerides and fatty acids, respectively. While chronic ICV infusions of OT (1.6 nmol/day) did not alter enzymes linked to triglyceride storage or lipogenesis (diacylglycerol *O*-acyltransferase homolog 1, fatty acid synthase, and acetyl-coenzyme A carboxylase alpha), it did stimulate enzymes associated with lipolysis (hormone-sensitive lipase and patatin-like phospholipase domain containing 2) (Deblon et al., 2011). Chronic CNS [ICV, 3V; 16 nmol/day] or systemic OT administration (1.6 mg/kg/day or ∼56.4 nmol/day) is also associated with decreases in respiratory quotient in DIO rats (Deblon et al., 2011; Blevins et al., 2016) and DIO mice (Maejima et al., 2011) relative to vehicle treated animals (Deblon et al., 2011; Maejima et al., 2011; Blevins et al., 2016) or pair-fed control animals (Deblon et al., 2011). Taken together, these findings suggest that OT-elicited lipolysis and lipid oxidation may contribute to OT-elicited weight loss.

## Oxytocin Receptor Dimerization

The OTR is a G protein-coupled receptor (GPCR) that is coupled to the Gαq alpha subunit (Gαq) or the Gαi alpha subunit (Busnelli and Chini, 2018). Given that the OTR is a GPCR it is prone to the formation of heterodimers with other GPCRs that are in close proximity. These heterodimers can impact intracellular signaling pathways, allosteric interactions, endocytosis, biological function and drug effects (Schellekens et al., 2013; Wallace Fitzsimons et al., 2019). This is of particular interest given that OT and ghrelin have opposing actions on food intake and OTRs and the growth hormone secretagogue receptor (GHS-R1a) have overlapping areas of expression in many CNS sites linked to the control of food intake and/or energy expenditure (including the ARC, VMH, NTS, and VTA) (Abizaid et al., 2006; Zigman et al., 2006). Recent studies have provided strong evidence for cross-talk between both receptors and co-expression of OTR/GHSR resulted in an attenuation of OTR-elicited signaling and potential heterocomplex formation (Wallace Fitzsimons et al., 2019).

In addition to ghrelin, the OTR has been found to form homo- and heterodimers with other receptors. OTRs and vasopressin V1a and V2 receptors also form homo- and heterodimers (Terrillon et al., 2003). In addition, OTR forms heterocomplexes with dopamine D2 receptors which may potentially contribute to anti-anxiety actions of OT within the CeA (de la Mora et al., 2016). Additional studies provide evidence for the presence of dopamine d2-OTR heteromers located within the ventral and dorsal striatum that may play a role with facilitatory receptor-receptor interactive effects (Romero-Fernandez et al., 2013). Recent studies also indicate that signaling through OT and serotonin 2A receptors appears to be impaired through heteroreceptor formation (Chruscicka et al., 2019). The extent to which these homo- or hetero-complexes may contribute to the effects of OT to reduce both anxiety and feeding reward and potentially explain the ability of OT to produce a more pronounced reduction of energy intake in DIO rodents (Deblon et al., 2011; Maejima et al., 2011; Blevins et al., 2016; Roberts et al., 2017; Edwards et al., 2021b) and obese humans (Thienel et al., 2016) will be important questions for future investigation.

## Does Oxytocin Reduce Body Weight in Obese and Overweight Humans?

The beneficial metabolic effects of OT have been recently translated to DIO non-human primates (Blevins et al., 2015) and obese humans (Zhang et al., 2013; Lawson et al., 2015; Thienel et al., 2016). Several small clinical trials conducted in normal weight or overweight/obese men have shown that a single-dose intranasal OT acutely decreased food intake and hedonic eating (Ott et al., 2013; Lawson et al., 2015; Thienel et al., 2016). Large clinical trials of long duration to examine the effectiveness of OT treatment in obesity is lacking but available clinical trial data have revealed beneficial effects of OT on reducing body weight in overweight/obese men and women as well as in an adolescent boy with hypothalamic obesity (Zhang et al., 2013; Hsu et al., 2017). In the first study to show that chronic intranasal OT elicited weight loss in humans, 24 overweight/obese men and women were randomized to receive OT nasal spray 24 international units (IU) four times daily or placebo for 8 weeks (Zhang et al., 2013). OT treatment led to a 8.9 ± 5.4 kg (*p* < 0.001) weight loss at the end of the trial and the weight reduction was significantly larger than the placebo at the end of the trial. No adverse events (AEs) were reported during the trial. In addition, in a single case study, chronic intranasal OT was found to reduce obesity and food intake in an obese 13-year-old adolescent following removal of a craniopharyngioma (Hsu et al., 2017), which speaks to the generalized energy homeostatic action of OT independent of obesity etiology. A more recent trial found that chronic intranasal OT (24 IU four times daily for 8 weeks) tended to reduce body adiposity and increase lean mass in a group of older adults with sarcopenia and obesity but failed to produce any changes in BMI (Espinoza et al., 2021). While these studies provided preliminary evidence that OT treatment may have promising metabolic benefits, including reduction of adiposity and/or body weight, sufficiently powered and rigorously designed clinical trials are still needed to confirm the effect of OT seen on energy metabolism. In addition to homeostatic food intake, OT has also been associated with limiting consumption of highly palatable foods in rodent and non-human primate models (for review, see Lawson, 2017; McCormack et al., 2020). These findings also have translated to some extent in humans (Lawson et al., 2015). Several labs have reported that intranasal OT reduced chocolate cookie or biscuit consumption (Ott et al., 2013; Thienel et al., 2016; Burmester et al., 2018). In addition, intranasal OT enhances the cognitive control of food craving in women who viewed images of candies and desserts (Striepens et al., 2016). To evaluate the mechanism for these behavioral findings, Plessow et al. (2018) observed that intranasal OT reduced activation of both homeostatic and feeding reward centers in the CNS, in response to images of highly palatable food during functional MRI. While further studies need to be done to clarify if OT modifies macronutrient preference in humans, these findings together suggest that it may play a role in limiting consumption of stress- or emotional-eating of food high in sugar or fat.

Thus, while several small clinical trials conducted in normal weight or overweight/obese men have now shown that a single-dose intranasal OT acutely decreased calorie intake (Lawson et al., 2015; Thienel et al., 2016), hedonic eating (Ott et al., 2013; Lawson et al., 2015; Thienel et al., 2016) and food craving (Striepens et al., 2016) it is still unclear if these findings would hold up in a paradigm of chronic administration or if they would be correlated with clinically meaningful weight loss. While the one study by Zhang et al. (2013) found that chronic intranasal OT elicited weight loss in overweight/obese humans, the mechanism of action of OT remains unexplored (e.g., associated with an increase in energy expenditure and/or decrease in appetite/food intake). Sufficiently powered, rigorously designed clinical trials are needed to examine the efficacy, tolerability and safety of OT use in humans. Based on our preclinical data and available clinical observations, we argue that OT holds promise as an appealing, non-invasive strategy to combat obesity though its CNS action on regulating appetite (Striepens et al., 2013; Freeman et al., 2016) and/or energy expenditure.

## Translational Potential

Intravenous/intramuscular OT is FDA-approved and has a long track record of clinical use in parturition (Vallera et al., 2017). Intranasal OT has been used clinically for over 50 years (most commonly in the field of psychiatry) and has an excellent safety profile (MacDonald et al., 2011). A systematic review of 38 RTCs conducted between 1990 and 2010 on over 1500 individuals (79% men) showed that OT was not associated with adverse outcomes when delivered at doses of 18–40 IU for short term use in controlled research settings (MacDonald et al., 2011). Mild side effects including drowsiness/sleepiness, calm/relaxed/comfortable, lightheadedness, and feeling of anxious/worried were reported by 279 out of 1529 participants (18%). Since then, longer term studies have been conducted and continued to demonstrate an excellent safety profile of intranasal OT (Tachibana et al., 2013; Zhang et al., 2013; Hsu et al., 2017; Cai et al., 2018). As mentioned earlier, the pharmacokinetics of intranasal OT administration have been investigated. Striepens et al. (2013) showed that, in subjects who received 24 IU of OT intranasally (or placebo), OT levels increased significantly in plasma at 15, 30, 45, and 60 min after administration, and in cerebrospinal fluid (CSF) at 75 min when the plasma level began to decline. In another study by Gossen et al. (2012), following 26 IU of single intranasal OT administration in eight men plasma OT concentrations increased over a period of 210 min, reaching a peak at 30 min. Lawson et al. (2015) previously showed that a single-dose (24 IU) intranasal OT acutely decreases food intake; Zhang et al. (2013) reported that 24 IU given QID over 8 weeks reduced body weight in overweight/obese men and women. The 40 IU dose has been used in clinical trials for up to approximately 10 weeks at a frequency of once daily and was well tolerated (van Zuiden et al., 2017; Flanagan et al., 2018).

As discussed above, OT may increase BAT thermogenesis through its action in the modulation of CNS sympathetic output and on sympathetic pre-ganglionic OTR-expressing neurons within the spinal cord to increase SNS outflow (see section “Source and Functions of Oxytocin”). Collectively, this may adversely affect cardiac function and hamper its translational potential (Greenfield et al., 2009; Kievit et al., 2013) given the high prevalence of hypertension and cardiovascular disease among individuals who are overweight or obese (Nguyen et al., 2008). However, studies in animals have generated mixed results demonstrating an increase, decrease or no change in blood pressure (Petersson et al., 1996; Nation et al., 2010; Maejima et al., 2011; Ludwig et al., 2013; Yosten and Samson, 2014) or heart rate (Petersson et al., 1996; Yang et al., 2009; Yoshida et al., 2009; Nation et al., 2010; Maejima et al., 2011; Ludwig et al., 2013; Hicks et al., 2014; Plante et al., 2015) depending on route of OT administration and species studied (for review, see Petersson, 2002; Gutkowska and Jankowski, 2012). Studies using intranasal OT in humans have not reported adverse side effects on heart rate (Burri et al., 2008; Kirkpatrick et al., 2014; Lawson et al., 2015), blood pressure (Kirkpatrick et al., 2014; Lawson et al., 2015) or cardiovascular dysfunction (Zhang et al., 2013) in men (Burri et al., 2008; Zhang et al., 2013; Kirkpatrick et al., 2014; Lawson et al., 2015) and non-pregnant women (Zhang et al., 2013; Kirkpatrick et al., 2014). The sample size of these studies was small, and the duration of treatment was short (up to 8 weeks). Therefore, it will be prudent to carefully monitor cardiovascular outcomes such as heart rate and blood pressure with chronic OT treatment in future studies.

## Conclusion

Pre-clinical data in rodents and non-human primates suggest that OT elicits weight loss, in part, by both central and peripheral mechanisms to reduce food intake (homeostatic and hedonic feeding) and impact energy expenditure in the absence of visceral illness or tolerance. While there is much enthusiasm over the potential use of OT as a therapeutic strategy to treat eating disorders and obesity, we await the results of ongoing clinical trials in obese humans for additional confirmation of its feasibility as a long-term weight loss strategy and assessment of adverse side effects. There is an ongoing need to identify an optimal dose, frequency, and duration of administration and to examine the dose-response effects of intranasal OT on body weight and adiposity in individuals with obesity.

Combination drug treatment has demonstrated impressive efficacy in inducing weight loss [(Frias et al., 2017; Chepurny et al., 2018)] (for review, see Rodgers et al., 2012). We think OT may be more optimal as an adjunct therapy for obesity rather than a monotherapy. It is clear that OT is effective as a monotherapy to elicit weight loss in DIO rodents (Deblon et al., 2011; Maejima et al., 2011, 2017; Zhang and Cai, 2011; Zhang et al., 2011; Morton et al., 2012; Blevins et al., 2016; Roberts et al., 2017; Edwards et al., 2021b), non-human primates (Blevins et al., 2015) and humans (Zhang et al., 2013), but these effects (pre- vs. post-intervention) appear to be modest even after sustained treatments that last 4–8 weeks [≈ 4.9% in DIO mice (Roberts et al., 2017), 8.7% in DIO rats (Roberts et al., 2017), 3.3% in DIO rhesus monkeys (Blevins et al., 2015) and 9.3% humans based on results from a proof of concept study with short duration and small sample size (Zhang et al., 2013)]. The average weight loss with the currently FDA approved medications (i.e., orlistat, liraglutide, naltrexone/bupropion, phentermine/topiramate, semaglutide) ranges between 5 and 14.9% (Allison et al., 2012; Apovian et al., 2015; Bray et al., 2018; Enebo et al., 2021).

Long-term efficacy of OT treatment for obesity remains to be established. The effect of OT on thermogenesis, appetite and stress reduction may offer unique properties that current therapies do not have. OT as an adjunct therapy for obesity is also worth exploring. For instance, pre-clinical and clinical studies indicate that OT in combination with the opioid antagonist, naltrexone, is an effective strategy to reduce food intake (animals, humans) as well as body weight (humans). Peripheral (intravenous) administration of OT and naltrexone was recently found to be effective at reducing palatable 10% sucrose solutions as well as intake of a high fat/high sugar diet in rats (Head et al., 2021). Intranasal OT was also found to be effective at reducing hyperphagia and maintaining weight loss when given in combination with the opioid antagonist, naltrexone, to a 13-year-old adolescent male with craniopharyngioma related hypothalamic obesity (Hsu et al., 2017). Thus, the combination of OT with other therapies that act, in part to reduce food intake (hedonic and/or homeostatic feeding) and increase energy expenditure may act in an additive mechanism to elicit greater weight loss than either treatment alone.

## Author Contributions

JN helped write and edit the manuscript and she also helped generate Figure 1. JT helped write and edit the manuscript. JB provided feedback on the figure and helped write and edit the manuscript. All authors contributed to the article and approved the submitted version.

## Conflict of Interest

JB has a financial interest in OXT Therapeutics, Inc., a company developing highly specific and stable analogs of oxytocin to treat obesity and metabolic disease. The authors’ interests were reviewed and are managed by their local institutions in accordance with their conflict of interest policies. The remaining authors declare that the research was conducted in the absence of any commercial or financial relationships that could be construed as a potential conflict of interest.

## Publisher’s Note

All claims expressed in this article are solely those of the authors and do not necessarily represent those of their affiliated organizations, or those of the publisher, the editors and the reviewers. Any product that may be evaluated in this article, or claim that may be made by its manufacturer, is not guaranteed or endorsed by the publisher.

## References

[B1] Abizaid A. Liu Z. W. Andrews Z. B. Shanabrough M. Borok E. Elsworth J. D. (2006). Ghrelin modulates the activity and synaptic input organization of midbrain dopamine neurons while promoting appetite. *J. Clin. Investig.* 116 3229–3239. 10.1172/JCI29867 17060947 PMC1618869

[B2] Allison D. B. Gadde K. M. Garvey W. T. Peterson C. A. Schwiers M. L. Najarian T. (2012). Controlled-release phentermine/topiramate in severely obese adults: a randomized controlled trial (EQUIP). *Obesity* 20 330–342. 10.1038/oby.2011.330 22051941 PMC3270297

[B3] Altirriba J. Poher A. L. Caillon A. Arsenijevic D. Veyrat-Durebex C. Lyautey J. (2014). Divergent effects of oxytocin treatment of obese diabetic mice on adiposity and diabetes. *Endocrinology* 155 4189–4201. 10.1210/en.2014-1466 25157455

[B4] Amico J. A. Vollmer R. R. Cai H. M. Miedlar J. A. Rinaman L. (2005). Enhanced initial and sustained intake of sucrose solution in mice with an oxytocin gene deletion. *Am. J. Physiol. Regul. Integr. Comp. Physiol.* 289 R1798–R1806. 16150836 10.1152/ajpregu.00558.2005

[B5] Angioni L. Cocco C. Ferri G. L. Argiolas A. Melis M. R. Sanna F. (2016). Involvement of nigral oxytocin in locomotor activity: a behavioral, immunohistochemical and lesion study in male rats. *Horm. Behav.* 83 23–38. 10.1016/j.yhbeh.2016.05.012 27189764

[B6] Antunes J. L. Zimmerman E. A. (1978). The hypothalamic magnocellular system of the rhesus monkey: an immunocytochemical study. *J. Comp. Neurol.* 181 539–565. 99459 10.1002/cne.901810306

[B7] Apovian C. M. Aronne L. J. Bessesen D. H. McDonnell M. E. Murad M. H. Pagotto U. (2015). Pharmacological management of obesity: an endocrine Society clinical practice guideline. *J. Clin. Endocrinol. Metab.* 100 342–362. 25590212 10.1210/jc.2014-3415

[B8] Arletti R. Benelli A. Bertolini A. (1989). Influence of oxytocin on feeding behavior in the rat. *Peptides* 10 89–93. 2748428 10.1016/0196-9781(89)90082-x

[B9] Arletti R. Benelli A. Bertolini A. (1990). Oxytocin inhibits food and fluid intake in rats. *Physiol. Behav.* 48 825–830. 2087513 10.1016/0031-9384(90)90234-u

[B10] Balazova L. Krskova K. Suski M. Sisovsky V. Hlavacova N. Olszanecki R. (2016). Metabolic effects of subchronic peripheral oxytocin administration in lean and obese zucker rats. *J. Physiol. Pharmacol.* 67 531–541. 27779474

[B11] Bales K. L. Perkeybile A. M. Conley O. G. Lee M. H. Guoynes C. D. Downing G. M. (2013). Chronic intranasal oxytocin causes long-term impairments in partner preference formation in male prairie voles. *Biol. Psychiatry* 74 180–188. 10.1016/j.biopsych.2012.08.025 23079235 PMC3556198

[B12] Banks W. A. DiPalma C. R. Farrell C. L. (1999). Impaired transport of leptin across the blood-brain barrier in obesity. *Peptides* 20 1341–1345. 10612449 10.1016/s0196-9781(99)00139-4

[B13] Bartz J. Simeon D. Hamilton H. Kim S. Crystal S. Braun A. (2011). Oxytocin can hinder trust and cooperation in borderline personality disorder. *Soc. Cogn. Affect. Neurosci.* 6 556–563. 21115541 10.1093/scan/nsq085 PMC3190211

[B14] Baskin D. G. Bastian L. S. (2010). “Immuno-laser capture microdissection of rat brain neruons for real time quantitative PCR,” in *Immunocytochemical methods and protocols, Methods in Molecular Biology*, eds Oliver C. Jamur M. C. (New York, NY: Humana Press), 219–230. 10.1007/978-1-59745-324-0_23 20012834

[B15] Baskin D. G. Kim F. Gelling R. W. Russell B. J. Schwartz M. W. Morton G. J. (2010). A new oxytocin-saporin cytotoxin for lesioning oxytocin-receptive neurons in the rat hindbrain. *Endocrinology* 151 4207–4213. 10.1210/en.2010-0295 20610562 PMC2940497

[B16] Beier K. T. Steinberg E. E. DeLoach K. E. Xie S. Miyamichi K. Schwarz L. (2015). Circuit architecture of VTA dopamine neurons revealed by systematic input-output mapping. *Cell* 162 622–634. 10.1016/j.cell.2015.07.015 26232228 PMC4522312

[B17] Benelli A. Bertolini A. Arletti R. (1991). Oxytocin-induced inhibition of feeding and drinking: no sexual dimorphism in rats. *Neuropeptides* 20 57–62. 10.1016/0143-4179(91)90040-p 1791926

[B18] Bergum D. Lonnee H. Hakli T. F. (2009). Oxytocin infusion: acute hyponatraemia, seizures and coma. *Acta Anaesth. Scand.* 53 826–827. 10.1111/j.1399-6576.2009.01964.x 19397503

[B19] Blevins J. E. Baskin D. G. (2015). Translational and therapeutic potential of oxytocin as an anti-obesity strategy: insights from rodents, nonhuman primates and humans. *Physiol. Behav.* 152 (Pt B), 438–449. 10.1016/j.physbeh.2015.05.023 26013577 PMC6235440

[B20] Blevins J. E. Eakin T. J. Murphy J. A. Schwartz M. W. Baskin D. G. (2003). Oxytocin innervation of caudal brainstem nuclei activated by cholecystokinin. *Brain Res.* 993 30–41. 10.1016/j.brainres.2003.08.036 14642828

[B21] Blevins J. E. Graham J. L. Morton G. J. Bales K. L. Schwartz M. W. Baskin D. G. (2015). Chronic oxytocin administration inhibits food intake, increases energy expenditure, and produces weight loss in fructose-fed obese rhesus monkeys. *Am. J. Physiol. Regul. Integr. Comp. Physiol.* 308 R431–R438. 10.1152/ajpregu.00441.2014 25540103 PMC4346756

[B22] Blevins J. E. Ho J. M. (2013). Role of oxytocin signaling in the regulation of body weight. *Rev. Endocrine Metab. Disord.* 14 311–329. 24065622 10.1007/s11154-013-9260-x PMC4213929

[B23] Blevins J. E. Morton G. J. Williams D. L. Caldwell D. W. Bastian L. S. Wisse B. E. (2009). Forebrain melanocortin signaling enhances the hindbrain satiety response to CCK-8. *Am. J. Physiol. Regul. Integr. Comp. Physiol.* 296 R476–R484. 10.1152/ajpregu.90544.2008 19109369 PMC3973398

[B24] Blevins J. E. Schwartz M. W. Baskin D. G. (2004). Evidence that paraventricular nucleus oxytocin neurons link hypothalamic leptin action to caudal brain stem nuclei controlling meal size. *Am. J. Physiol. Regul. Integr. Comp. Physiol.* 287 R87–R96. 10.1152/ajpregu.00604.2003 15044184

[B25] Blevins J. E. Thompson B. W. Anekonda V. T. Ho J. M. Graham J. L. Roberts Z. S. (2016). Chronic CNS oxytocin signaling preferentially induces fat loss in high fat diet-fed rats by enhancing satiety responses and increasing lipid utilization. *Am. J. Physiol. Reg. I* 310 R640–R58. 10.1152/ajpregu.00220.2015 26791828 PMC4867381

[B26] Blouet C. Jo Y. H. Li X. Schwartz G. J. (2009). Mediobasal hypothalamic leucine sensing regulates food intake through activation of a hypothalamus-brainstem circuit. *J. Neurosci.* 29 8302–8311. 10.1523/JNEUROSCI.1668-09.2009 19571121 PMC2740923

[B27] Boccia M. L. Panicker A. K. Pedersen C. Petrusz P. (2001). Oxytocin receptors in non-human primate brain visualized with monoclonal antibody. *Neuroreport* 12 1723–1726. 10.1097/00001756-200106130-00041 11409747

[B28] Boccia M. L. Petrusz P. Suzuki K. Marson L. Pedersen C. A. (2013). Immunohistochemical localization of oxytocin receptors in human brain. *Neuroscience* 253 155–164. 24012742 10.1016/j.neuroscience.2013.08.048

[B29] Borg J. Simren M. Ohlsson B. (2011). Oxytocin reduces satiety scores without affecting the volume of nutrient intake or gastric emptying rate in healthy subjects. *Neurogastroenterol. Motil.* 23 56.e5–61.e5. 20868426 10.1111/j.1365-2982.2010.01599.x

[B30] Born J. Lange T. Kern W. McGregor G. P. Bickel U. Fehm H. L. (2002). Sniffing neuropeptides: a transnasal approach to the human brain. *Nat. Neurosci.* 5 514–516. 10.1038/nn849 11992114

[B31] Braude R. Mitchell K. G. (1952). Observations on the relationship between oxytocin and adrenaline in milk ejection in the sow. *J. Endocrinol.* 8 238–241. 10.1677/joe.0.0080238 14955555

[B32] Bray G. A. Heisel W. E. Afshin A. Jensen M. D. Dietz W. H. Long M. (2018). The science of obesity management: an endocrine society scientific statement. *Endocr. Rev.* 39 79–132. 29518206 10.1210/er.2017-00253 PMC5888222

[B33] Breuil V. Amri E. Z. Panaia-Ferrari P. Testa J. Elabd C. Albert-Sabonnadiere C. (2011). Oxytocin and bone remodelling: relationships with neuropituitary hormones, bone status and body composition. *Joint Bone Spine* 78 611–615. 10.1016/j.jbspin.2011.02.002 21441053

[B34] Breuil V. Fontas E. Chapurlat R. Panaia-Ferrari P. Yahia H. B. Faure S. (2015). Oxytocin and bone status in men: analysis of the MINOS cohort. *Osteoporos. Int.* 26 2877–2882. 10.1007/s00198-015-3201-3 26109496

[B35] Breuil V. Panaia-Ferrari P. Fontas E. Roux C. Kolta S. Eastell R. (2014). Oxytocin, a new determinant of bone mineral density in post-menopausal women: analysis of the OPUS cohort. *J. Clin. Endocrinol. Metab.* 99 E634–E641. 10.1210/jc.2013-4126 24446658

[B36] Brierley D. I. Holt M. K. Singh A. de Araujo A. McDougle M. Vergara M. (2021). Central and peripheral GLP-1 systems independently suppress eating. *Nat. Metab.* 3 258–273. 33589843 10.1038/s42255-021-00344-4 PMC7116821

[B37] Brownstein M. J. Russell J. T. Gainer H. (1980). Synthesis, transport, and release of posterior pituitary hormones. *Science* 207 373–378. 6153132 10.1126/science.6153132

[B38] Burmester V. Higgs S. Terry P. (2018). Rapid-onset anorectic effects of intranasal oxytocin in young men. *Appetite* 130 104–109. 10.1016/j.appet.2018.08.003 30081055

[B39] Burri A. Heinrichs M. Schedlowski M. Kruger T. H. (2008). The acute effects of intranasal oxytocin administration on endocrine and sexual function in males. *Psychoneuroendocrinology* 33 591–600. 10.1016/j.psyneuen.2008.01.014 18375074

[B40] Busnelli M. Chini B. (2018). Molecular basis of oxytocin receptor signalling in the brain: what we know and what we need to know. *Curr. Top. Behav. Neurosci.* 35 3–29. 10.1007/7854_2017_6 28812263

[B41] Cai Q. Feng L. Yap K. Z. (2018). Systematic review and meta-analysis of reported adverse events of long-term intranasal oxytocin treatment for autism spectrum disorder. *Psychiatry Clin. Neurosci.* 72 140–151. 10.1111/pcn.12627 29232031

[B42] Caldwell J. D. Jirikowski G. F. Greer E. R. Stumpf W. E. Pedersen C. A. (1988). Ovarian steroids and sexual interaction alter oxytocinergic content and distribution in the basal forebrain. *Brain Res.* 446 236–244. 10.1016/0006-8993(88)90882-7 3370488

[B43] Camerino C. (2009). Low sympathetic tone and obese phenotype in oxytocin-deficient mice. *Obesity* 17 980–984. 10.1038/oby.2009.12 19247273

[B44] Cannon B. Nedergaard J. (2004). Brown adipose tissue: function and physiological significance. *Physiol. Rev.* 84 277–359. 14715917 10.1152/physrev.00015.2003

[B45] Carson D. S. Hunt G. E. Guastella A. J. Barber L. Cornish J. L. Arnold J. C. (2010). Systemically administered oxytocin decreases methamphetamine activation of the subthalamic nucleus and accumbens core and stimulates oxytocinergic neurons in the hypothalamus. *Addict. Biol.* 15 448–463. 10.1111/j.1369-1600.2010.00247.x 20731630

[B46] Castel M. Morris J. F. (1988). The neurophysin-containing innervation of the forebrain of the mouse. *Neuroscience* 24 937–966. 10.1016/0306-4522(88)90078-4 3380308

[B47] Cechetto D. F. Saper C. B. (1988). Neurochemical organization of the hypothalamic projection to the spinal cord in the rat. *J. Comp. Neurol.* 272 579–604. 10.1002/cne.902720410 2901438

[B48] Chang S. W. Platt M. L. (2014). Oxytocin and social cognition in rhesus macaques: implications for understanding and treating human psychopathology. *Brain Res.* 1580 57–68. 10.1016/j.brainres.2013.11.006 24231551 PMC4017005

[B49] Chepurny O. G. Bonaccorso R. L. Leech C. A. Wollert T. Langford G. M. Schwede F. (2018). Chimeric peptide EP45 as a dual agonist at GLP-1 and NPY2R receptors. *Sci. Rep.* 8:3749. 10.1038/s41598-018-22106-1 PMC5830615 29491394

[B50] Chruscicka B. Wallace Fitzsimons S. E. Borroto-Escuela D. O. Druelle C. Stamou P. Nally K. (2019). Attenuation of oxytocin and serotonin 2A receptor signaling through novel heteroreceptor formation. *ACS Chem. Neurosci.* 10 3225–3240. 10.1021/acschemneuro.8b00665 31038917

[B51] Colaianni G. Sun L. Benedetto A. Di Tamma R. Zhu L. L. Cao J. (2012). Bone marrow oxytocin mediates the anabolic action of estrogen on the skeleton. *J. Biol. Chem.* 287 29159–29167. 10.1074/jbc.M112.365049 22761429 PMC3436530

[B52] Colaianni G. Sun L. Zaidi M. Zallone A. (2015). The “love hormone” oxytocin regulates the loss and gain of the fat-bone relationship. *Front. Endocrinol.* 6:79. 10.3389/fendo.2015.00079 26042088 PMC4435037

[B53] Colucci S. Colaianni G. Mori G. Grano M. Zallone A. (2002). Human osteoclasts express oxytocin receptor. *Biochem. Biophys. Res. Commun.* 297 442–445. 12270111 10.1016/s0006-291x(02)02009-0

[B54] Copland J. A. Ives K. L. Simmons D. J. Soloff M. S. (1999). Functional oxytocin receptors discovered in human osteoblasts. *Endocrinology* 140 4371–4374. 10.1210/endo.140.9.7130 10465312

[B55] Cornier M. A. Dabelea D. Hernandez T. L. Lindstrom R. C. Steig A. J. Stob N. R. (2008). The metabolic syndrome. *Endocrine Rev.* 29 777–822. 18971485 10.1210/er.2008-0024 PMC5393149

[B56] Daniels D. Flanagan-Cato L. M. (2000). Functionally-defined compartments of the lordosis neural circuit in the ventromedial hypothalamus in female rats. *J. Neurobiol.* 45 1–13. 10992252

[B57] de la Mora M. P. Perez-Carrera D. Crespo-Ramirez M. Tarakanov A. Fuxe K. Borroto-Escuela D. O. (2016). Signaling in dopamine D2 receptor-oxytocin receptor heterocomplexes and its relevance for the anxiolytic effects of dopamine and oxytocin interactions in the amygdala of the rat. *Biochim. Biophys. Acta* 1862 2075–2085. 10.1016/j.bbadis.2016.07.004 27425032

[B58] Deblon N. Veyrat-Durebex C. Bourgoin L. Caillon A. Bussier A. L. Petrosino S. (2011). Mechanisms of the anti-obesity effects of oxytocin in diet-induced obese rats. *PLoS One* 6:e25565. 10.1371/journal.pone.0025565 21980491 PMC3181274

[B59] den Hertog C. E. de Groot A. N. van Dongen P. W. (2001). History and use of oxytocics. *Eur. J. Obstetr. Gynecol. Reproduct. Biol.* 94 8–12. 10.1016/s0301-2115(00)00311-0 11134819

[B60] Dierickx K. Vandesande F. (1977). Immunocytochemical localization of the vasopressinergic and the oxytocinergic neurons in the human hypothalamus. *Cell Tissue Res.* 184 15–27. 10.1007/BF00220524 336217

[B61] Eckel R. H. Grundy S. M. Zimmet P. Z. (2005). The metabolic syndrome. *Lancet* 365 1415–1428. 15836891 10.1016/S0140-6736(05)66378-7

[B62] Eckertova M. Ondrejcakova M. Krskova K. Zorad S. Jezova D. (2011). Subchronic treatment of rats with oxytocin results in improved adipocyte differentiation and increased gene expression of factors involved in adipogenesis. *Br. J. Pharmacol.* 162 452–463. 10.1111/j.1476-5381.2010.01037.x 20846187 PMC3031065

[B63] Edwards M. M. Nguyen H. K. Herbertson A. J. Dodson A. D. Wietecha T. Wolden-Hanson T. (2021b). Chronic hindbrain administration of oxytocin elicits weight loss in male diet-induced obese mice. *Am. J. Physiol. Regul. Integr. Comp. Physiol.* 320 R471–R487. 33470901 10.1152/ajpregu.00294.2020 PMC8238148

[B64] Edwards M. M. Nguyen H. K. Dodson A. D. Herbertson A. J. Wietecha T. A. Wolden-Hanson T. (2021a). Effects of combined oxytocin and beta-3 receptor agonist (CL 316243) treatment on body weight and adiposity in male diet-induced obese rats. *Front. Physiol.* 12:725912. 10.3389/fphys.2021.725912 34566687 PMC8457402

[B65] Elabd C. Cousin W. Upadhyayula P. Chen R. Y. Chooljian M. S. Li J. (2014). Oxytocin is an age-specific circulating hormone that is necessary for muscle maintenance and regeneration. *Nat. Commun.* 5:4082. 10.1038/ncomms5082 24915299 PMC4512838

[B66] Enebo L. B. Berthelsen K. K. Kankam M. Lund M. T. Rubino D. M. Satylganova A. (2021). Safety, tolerability, pharmacokinetics, and pharmacodynamics of concomitant administration of multiple doses of cagrilintide with semaglutide 2.4 mg for weight management: a randomised, controlled, phase 1b trial. *Lancet* 397 1736–1748. 10.1016/S0140-6736(21)00845-X 33894838

[B67] Erdenebayar O. Kato T. Kawakita T. Kasai K. Kadota Y. Yoshida K. (2020). Effects of peripheral oxytocin administration on body weight, food intake, adipocytes, and biochemical parameters in peri- and postmenopausal female rats. *Endocrine J.* 68 7–16. 10.1507/endocrj.EJ19-0586 32879161

[B68] Ermisch A. Barth T. Ruhle H. J. Skopkova J. Hrbas P. Landgraf R. (1985). On the blood-brain barrier to peptides: accumulation of labelled vasopressin, DesGlyNH2-vasopressin and oxytocin by brain regions. *Endocrinol. Exp.* 19 29–37. 3872788

[B69] Espinoza S. E. Lee J. L. Wang C. P. Ganapathy V. MacCarthy D. Pascucci C. (2021). Intranasal oxytocin improves lean muscle mass and lowers LDL cholesterol in older adults with sarcopenic obesity: a pilot randomized controlled trial. *J. Am. Med. Dir. Assoc.* 22 1877.e2–1882.e2. 10.1016/j.jamda.2021.04.015 34029521 PMC8567747

[B70] Fenselau H. Campbell J. N. Verstegen A. M. Madara J. C. Xu J. Shah B. P. (2017). A rapidly acting glutamatergic ARC–>PVH satiety circuit postsynaptically regulated by alpha-MSH. *Nat. Neurosci.* 20 42–51. 10.1038/nn.4442 27869800 PMC5191921

[B71] Finkelstein E. A. Trogdon J. G. Cohen J. W. Dietz W. (2009). Annual medical spending attributable to obesity: payer-and service-specific estimates. *Health Affairs* 28 w822–w831. 10.1377/hlthaff.28.5.w822 19635784

[B72] Flanagan J. C. Sippel L. M. Wahlquist A. Moran-Santa Maria M. M. Back S. E. (2018). Augmenting Prolonged Exposure therapy for PTSD with intranasal oxytocin: a randomized, placebo-controlled pilot trial. *J. Psychiatr. Res.* 98 64–69. 10.1016/j.jpsychires.2017.12.014 29294429 PMC5800951

[B73] Flanagan L. M. Olson B. R. Sved A. F. Verbalis J. G. Stricker E. M. (1992). Gastric motility in conscious rats given oxytocin and an oxytocin antagonist centrally. *Brain Res.* 578 256–260. 10.1016/0006-8993(92)90255-8 1324762

[B74] Flanagan-Cato L. M. Calizo L. H. Daniels D. (2001). The synaptic organization of VMH neurons that mediate the effects of estrogen on sexual behavior. *Hormon. Behav.* 40 178–182. 10.1006/hbeh.2001.1679 11534979

[B75] Fosgerau K. Raun K. Nilsson C. Dahl K. Wulff B. S. (2014). Novel alpha-MSH analog causes weight loss in obese rats and minipigs and improves insulin sensitivity. *J. Endocrinol.* 220 97–107. 10.1530/JOE-13-0284 24204009 PMC3888513

[B76] Freeman S. M. Inoue K. Smith A. L. Goodman M. M. Young L. J. (2014). The neuroanatomical distribution of oxytocin receptor binding and mRNA in the male rhesus macaque (*Macaca mulatta*). *Psychoneuroendocrinology* 45 128–141. 10.1016/j.psyneuen.2014.03.023 24845184 PMC4043226

[B77] Freeman S. M. Ngo J. Singh B. Masnaghetti M. Bales K. L. Blevins J. E. (2018). Effects of chronic oxytocin administration and diet composition on oxytocin and vasopressin 1a receptor binding in the rat brain. *Neuroscience* 392 241–251. 10.1016/j.neuroscience.2018.07.037 30071278 PMC6204308

[B78] Freeman S. M. Samineni S. Allen P. C. Stockinger D. Bales K. L. Hwa G. G. (2016). Plasma and CSF oxytocin levels after intranasal and intravenous oxytocin in awake macaques. *Psychoneuroendocrinology* 66 185–194. 10.1016/j.psyneuen.2016.01.014 26826355

[B79] Freund-Mercier M. J. Stoeckel M. E. (1995). Somatodendritic autoreceptors on oxytocin neurones. *Adv. Exp. Med. Biol.* 395 185–194. 8713963

[B80] Freund-Mercier M. J. Stoeckel M. E. Klein M. J. (1994). Oxytocin receptors on oxytocin neurones: histoautoradiographic detection in the lactating rat. *J. Physiol.* 480(Pt 1), 155–161. 7853219 10.1113/jphysiol.1994.sp020349 PMC1155786

[B81] Frias J. P. Bastyr E. J. Vignati L. Tschop M. H. Schmitt C. Owen K. (2017). The sustained effects of a dual GIP/GLP-1 receptor agonist, NNC0090-2746, in patients with Type 2 diabetes. *Cell Metab.* 26 343–345. 10.1016/j.cmet.2017.07.011 28768173

[B82] Gajdosechova L. Krskova K. Olszanecki R. Zorad S. (2015). Differential regulation of oxytocin receptor in various adipose tissue depots and skeletal muscle types in obese Zucker rats. *Hormone Metab. Res.* 47 600–604. 10.1055/s-0034-1395677 25565097

[B83] Gajdosechova L. Krskova K. Segarra A. B. Spolcova A. Suski M. Olszanecki R. (2014). Hypooxytocinaemia in obese Zucker rats relates to oxytocin degradation in liver and adipose tissue. *J. Endocrinol.* 220 333–343. 10.1530/JOE-13-0417 24389591

[B84] Gao F. Zheng K. I. Wang X. B. Sun Q. F. Pan K. H. Wang T. Y. (2020). Obesity is a risk factor for greater COVID-19 severity. *Diabetes Care* 43 e72–e74. 32409499 10.2337/dc20-0682

[B85] George J. M. (1978). Immunoreactive vasopressin and oxytocin: concentration in individual human hypothalamic nuclei. *Science* 200 342–343. 10.1126/science.556308 556308

[B86] Gimpl G. Fahrenholz F. (2001). The oxytocin receptor system: structure, function, and regulation. *Physiol. Rev.* 81 629–683. 11274341 10.1152/physrev.2001.81.2.629

[B87] Ginsberg S. D. Hof P. R. Young W. G. Morrison J. H. (1994). Noradrenergic innervation of vasopressin- and oxytocin-containing neurons in the hypothalamic paraventricular nucleus of the macaque monkey: quantitative analysis using double-label immunohistochemistry and confocal laser microscopy. *J. Comp. Neurol.* 341 476–491. 10.1002/cne.903410405 8201025

[B88] Gossen A. Hahn A. Westphal L. Prinz S. Schultz R. T. Grunder G. (2012). Oxytocin plasma concentrations after single intranasal oxytocin administration - a study in healthy men. *Neuropeptides* 46 211–215. 10.1016/j.npep.2012.07.001 22884888

[B89] Gould B. R. Zingg H. H. (2003). Mapping oxytocin receptor gene expression in the mouse brain and mammary gland using an oxytocin receptor-LacZ reporter mouse. *Neuroscience* 122 155–167. 10.1016/s0306-4522(03)00283-5 14596857

[B90] Gouzenes L. Desarmenien M. G. Hussy N. Richard P. Moos F. C. (1998). Vasopressin regularizes the phasic firing pattern of rat hypothalamic magnocellular vasopressin neurons. *J. Neurosci.* 18 1879–1885. 10.1523/JNEUROSCI.18-05-01879.1998 9465012 PMC6792631

[B91] Greenfield J. R. Miller J. W. Keogh J. M. Henning E. Satterwhite J. H. Cameron G. S. (2009). Modulation of blood pressure by central melanocortinergic pathways. *N. Engl. J. Med.* 360 44–52. 19092146 10.1056/NEJMoa0803085

[B92] Grinevich V. Neumann I. D. (2020). Brain oxytocin: how puzzle stones from animal studies translate into psychiatry. *Mol. Psychiatry* 26 265–279. 10.1038/s41380-020-0802-9 32514104 PMC7278240

[B93] Grundy S. M. (2008). Metabolic syndrome pandemic. *Arterioscler. Thromb. Vasc. Biol.* 28 629–636. 18174459 10.1161/ATVBAHA.107.151092

[B94] Guo W. Li M. Dong Y. Zhou H. Zhang Z. Tian C. (2020). Diabetes is a risk factor for the progression and prognosis of COVID-19. *Diabetes Metab. Res. Rev.* [Epub ahead of print]. 10.1002/dmrr.3319 PMC7228407 32233013

[B95] Gutkowska J. Jankowski M. (2012). Oxytocin revisited: its role in cardiovascular regulation. *J. Neuroendocrinol.* 24 599–608. 21981277 10.1111/j.1365-2826.2011.02235.x

[B96] Hales C. M. Carroll M. D. Fryar C. D. Ogden C. L. (2020). Prevalence of obesity and severe obesity among adults: United States, 2017-2018. *NCHS Data Brief* 360 1–8. 32487284

[B97] Hallbeck M. Larhammar D. Blomqvist A. (2001). Neuropeptide expression in rat paraventricular hypothalamic neurons that project to the spinal cord. *J. Comp. Neurol.* 433 222–238. 11283961 10.1002/cne.1137

[B98] Halseth K. Shan Gilder K. Malone M. Acevedo L. Fujioka K. (2018). Quality of life, binge eating and sexual function in participants treated for obesity with sustained release naltrexone/bupropion. *Obes. Sci. Pract.* 4 141–152. 10.1002/osp4.156 29670752 PMC5893468

[B99] Hayashi R. Kasahara Y. Hidema S. Fukumitsu S. Nakagawa K. Nishimori K. (2020). Oxytocin ameliorates impaired behaviors of high fat diet-induced obese mice. *Front. Endocrinol.* 11:379. 10.3389/fendo.2020.00379 32719656 PMC7347791

[B100] Head M. A. Levine A. S. Christian D. G. Klockars A. Olszewski P. K. (2021). Effect of combination of peripheral oxytocin and naltrexone at subthreshold doses on food intake, body weight and feeding-related brain gene expression in male rats. *Physiol. Behav.* 238:113464. 10.1016/j.physbeh.2021.113464 34022256

[B101] Herisson F. M. Waas J. R. Fredriksson R. Schioth H. B. Levine A. S. Olszewski P. K. (2016). Oxytocin acting in the nucleus accumbens core decreases food intake. *J. Neuroendocrinol.* 28:12381. 10.1111/jne.12381 27114001

[B102] Hicks C. Jorgensen W. Brown C. Fardell J. Koehbach J. Gruber C. W. (2012). The nonpeptide oxytocin receptor agonist WAY 267,464: receptor-binding profile, prosocial effects and distribution of c-Fos expression in adolescent rats. *J. Neuroendocrinol.* 24 1012–1029. 10.1111/j.1365-2826.2012.02311.x 22420322 PMC3399775

[B103] Hicks C. Ramos L. Reekie T. Misagh G. H. Narlawar R. Kassiou M. (2014). Body temperature and cardiac changes induced by peripherally administered oxytocin, vasopressin and the non-peptide oxytocin receptor agonist WAY 267,464: a biotelemetry study in rats. *Br. J. Pharmacol.* 171 2868–2887. 10.1111/bph.12613 24641248 PMC4243861

[B104] Hidema S. Fukuda T. Hiraoka Y. Mizukami H. Hayashi R. Otsuka A. (2016). Generation of Oxtr cDNA(HA) -Ires-Cre mice for gene expression in an oxytocin receptor specific manner. *J. Cell. Biochem.* 117 1099–1111. 10.1002/jcb.25393 26442453

[B105] Ho J. M. Anekonda V. T. Thompson B. W. Zhu M. Curry R. W. Hwang B. H. (2014). Hindbrain oxytocin receptors contribute to the effects of circulating oxytocin on food intake in male rats. *Endocrinology* 155 2845–2857. 10.1210/en.2014-1148 24877632 PMC4098005

[B106] Hollander P. Gupta A. K. Plodkowski R. Greenway F. Bays H. Burns C. (2013). Effects of naltrexone sustained-release/bupropion sustained-release combination therapy on body weight and glycemic parameters in overweight and obese patients with type 2 diabetes. *Diabetes Care* 36 4022–4029. 10.2337/dc13-0234 24144653 PMC3836105

[B107] Hsu E. A. Miller J. L. Perez F. A. Roth C. L. (2017). Oxytocin and Naltrexone successfully treat hypothalamic obesity in a boy post-craniopharyngioma resection. *J. Clin. Endocrinol. Metab.* 103 370–l375. 10.1210/jc.2017-02080 29220529

[B108] Insel T. R. Winslow J. T. Witt D. M. (1992). Homologous regulation of brain oxytocin receptors. *Endocrinology* 130 2602–2608. 1315251 10.1210/endo.130.5.1315251

[B109] Iwasa T. Matsuzaki T. Mayila Y. Yanagihara R. Yamamoto Y. Kawakita T. (2019b). Oxytocin treatment reduced food intake and body fat and ameliorated obesity in ovariectomized female rats. *Neuropeptides* 75 49–57. 10.1016/j.npep.2019.03.002 30885500

[B110] Iwasa T. Matsuzaki T. Mayila Y. Kawakita T. Yanagihara R. Irahara M. (2019a). The effects of chronic oxytocin administration on body weight and food intake in DHT-induced PCOS model rats. *Gynecol. Endocrinol.* 36 55–60. 31220962 10.1080/09513590.2019.1631276

[B111] Iwasa T. Matsuzaki T. Mayila Y. Kawakita T. Yanagihara R. Irahara M. (2020). The effects of chronic oxytocin administration on body weight and food intake in DHT-induced PCOS model rats. *Gynecol. Endocrinol.* 36 55–60. 31220962 10.1080/09513590.2019.1631276

[B112] Iwasaki Y. Kumari P. Wang L. Hidema S. Nishimori K. Yada T. (2019). Relay of peripheral oxytocin to central oxytocin neurons via vagal afferents for regulating feeding. *Biochem. Biophys. Res. Commun.* 519 553–558. 10.1016/j.bbrc.2019.09.039 31537381

[B113] Iwasaki Y. Maejima Y. Suyama S. Yoshida M. Arai T. Katsurada K. (2014). Peripheral oxytocin activates vagal afferent neurons to suppress feeding in normal and leptin-resistant mice: a route for ameliorating hyperphagia and obesity. *Am. J. Physiol. Regul. Integr. Comp. Physiol.* 308 R360–R369. 10.1152/ajpregu.00344.2014 25540101

[B114] Iwasaki Y. Maejima Y. Suyama S. Yoshida M. Arai T. Katsurada K. (2015). Peripheral oxytocin activates vagal afferent neurons to suppress feeding in normal and leptin-resistant mice: a route for ameliorating hyperphagia and obesity. *Am. J. Physiol. Regul. Integr. Comp. Physiol.* 308 R360–R369. 25540101 10.1152/ajpregu.00344.2014

[B115] Jankowski M. Hajjar F. Kawas S. A. Mukaddam-Daher S. Hoffman G. McCann S. M. (1998). Rat heart: a site of oxytocin production and action. *Proc. Natl. Acad. Sci. U.S.A.* 95 14558–14563. 10.1073/pnas.95.24.14558 9826739 PMC24412

[B116] Jansen A. S. Wessendorf M. W. Loewy A. D. (1995). Transneuronal labeling of CNS neuropeptide and monoamine neurons after pseudorabies virus injections into the stellate ganglion. *Brain Res.* 683 1–24. 10.1016/0006-8993(95)00276-v 7552333

[B117] Jirikowski G. F. Caldwell J. D. Pedersen C. A. Stumpf W. E. (1988). Estradiol influences oxytocin-immunoreactive brain systems. *Neuroscience* 25 237–248. 10.1016/0306-4522(88)90022-x 3393280

[B118] Johnstone L. E. Fong T. M. Leng G. (2006). Neuronal activation in the hypothalamus and brainstem during feeding in rats. *Cell Metab.* 4 313–321. 17011504 10.1016/j.cmet.2006.08.003

[B119] Jordan R. E. Adab P. Cheng K. K. (2020). Covid-19: risk factors for severe disease and death. *BMJ* 368:m1198. 10.1136/bmj.m1198 32217618

[B120] Kasahara Y. Sato K. Takayanagi Y. Mizukami H. Ozawa K. Hidema S. (2013). Oxytocin receptor in the hypothalamus is sufficient to rescue normal thermoregulatory function in male oxytocin receptor knockout mice. *Endocrinology* 154 4305–4315. 10.1210/en.2012-2206 24002032

[B121] Kasahara Y. Takayanagi Y. Kawada T. Itoi K. Nishimori K. (2007). Impaired thermoregulatory ability of oxytocin-deficient mice during cold-exposure. *Biosci. Biotechnol. Biochem.* 71 3122–3126. 10.1271/bbb.70498 18071238

[B122] Kasahara Y. Tateishi Y. Hiraoka Y. Otsuka A. Mizukami H. Ozawa K. (2015). Role of the oxytocin receptor expressed in the rostral medullary raphe in thermoregulation during cold conditions. *Front. Endocrinol.* 6:180. 10.3389/fendo.2015.00180 26635729 PMC4658403

[B123] Kataoka N. Hioki H. Kaneko T. Nakamura K. (2014). Psychological stress activates a dorsomedial hypothalamus-medullary raphe circuit driving brown adipose tissue thermogenesis and hyperthermia. *Cell Metab.* 20 346–358. 10.1016/j.cmet.2014.05.018 24981837

[B124] Kawata M. Sano Y. (1982). Immunohistochemical identification of the oxytocin and vasopressin neurons in the hypothalamus of the monkey (*Macaca fuscata*). *Anatom. Embryol.* 165 151–167. 10.1007/BF00305474 7158807

[B125] Kendrick K. M. (2000). Oxytocin, motherhood and bonding. *Exp. Physiol.* 85 111S–124S. 10.1111/j.1469-445x.2000.tb00014.x 10795913

[B126] Kendrick K. M. Keverne E. B. Baldwin B. A. Sharman D. F. (1986). Cerebrospinal fluid levels of acetylcholinesterase, monoamines and oxytocin during labour, parturition, vaginocervical stimulation, lamb separation and suckling in sheep. *Neuroendocrinology* 44 149–156. 10.1159/000124638 3796790

[B127] Kievit P. Halem H. Marks D. L. Dong J. Z. Glavas M. M. Sinnayah P. (2013). Chronic treatment with a melanocortin-4 receptor agonist causes weight loss, reduces insulin resistance, and improves cardiovascular function in diet-induced obese rhesus macaques. *Diabetes* 62 490–497. 10.2337/db12-0598 23048186 PMC3554387

[B128] Kirchgessner A. L. Sclafani A. (1988). PVN-hindbrain pathway involved in the hypothalamic hyperphagia-obesity syndrome. *Physiol. Behav.* 42 517–528. 3166142 10.1016/0031-9384(88)90153-9

[B129] Kirchgessner A. L. Sclafani A. Nilaver G. (1988). Histochemical identification of a PVN-hindbrain feeding pathway. *Physiol. Behav.* 42 529–543. 10.1016/0031-9384(88)90154-0 2842813

[B130] Kirkpatrick M. G. Francis S. M. Lee R. de Wit H. Jacob S. (2014). Plasma oxytocin concentrations following MDMA or intranasal oxytocin in humans. *Psychoneuroendocrinology* 46 23–31. 24882155 10.1016/j.psyneuen.2014.04.006 PMC4088952

[B131] Klockars A. Brunton C. Li L. Levine A. S. Olszewski P. K. (2017). Intravenous administration of oxytocin in rats acutely decreases deprivation-induced chow intake, but it fails to affect consumption of palatable solutions. *Peptides* 93 13–19. 10.1016/j.peptides.2017.04.010 28460894

[B132] Klockars A. Levine A. S. Olszewski P. K. (2015). Central oxytocin and food intake: focus on macronutrient-driven reward. *Front. Endocrinol.* 6:65. 10.3389/fendo.2015.00065 25972841 PMC4412129

[B133] Klockars O. A. Klockars A. Levine A. S. Olszewski P. K. (2018). Oxytocin administration in the basolateral and central nuclei of amygdala moderately suppresses food intake. *Neuroreport* 29 504–510. 10.1097/WNR.0000000000001005 29538098

[B134] Klockars O. A. Waas J. R. Klockars A. Levine A. S. Olszewski P. K. (2017). Neural basis of ventromedial hypothalamic oxytocin-driven decrease in appetite. *Neuroscience* 366 54–61. 10.1016/j.neuroscience.2017.10.008 29037599

[B135] Knobloch H. S. Charlet A. Hoffmann L. C. Eliava M. Khrulev S. Cetin A. H. (2012). Evoked axonal oxytocin release in the central amygdala attenuates fear response. *Neuron* 73 553–566. 10.1016/j.neuron.2011.11.030 22325206

[B136] Koutcherov Y. Mai J. K. Ashwell K. W. Paxinos G. (2000). Organization of the human paraventricular hypothalamic nucleus. *J. Comp. Neurol.* 423 299–318. 10867660

[B137] Kublaoui B. M. Gemelli T. Tolson K. P. Wang Y. Zinn A. R. (2008). Oxytocin deficiency mediates hyperphagic obesity of Sim1 haploinsufficient mice. *Mol. Endocrinol.* 22 1723–1734. 10.1210/me.2008-0067 18451093 PMC2453606

[B138] Kusminski C. M. Bickel P. E. Scherer P. E. (2016). Targeting adipose tissue in the treatment of obesity-associated diabetes. *Nat. Rev. Drug Discov.* 15 639–660. 27256476 10.1038/nrd.2016.75

[B139] Labyb M. Chretien C. Caillon A. Rohner-Jeanrenaud F. Altirriba J. (2018). Oxytocin administration alleviates acute but not chronic leptin resistance of diet-induced obese mice. *Int. J. Mol. Sci.* 20:88. 10.3390/ijms20010088 PMC6337181 30587816

[B140] Lawson E. A. (2017). The effects of oxytocin on eating behaviour and metabolism in humans. *Nat. Rev. Endocrinol.* 13 700–709. 28960210 10.1038/nrendo.2017.115 PMC5868755

[B141] Lawson E. A. Marengi D. A. DeSanti R. L. Holmes T. M. Schoenfeld D. A. Tolley C. J. (2015). Oxytocin reduces caloric intake in men. *Obesity* 23 950–956. 25865294 10.1002/oby.21069 PMC4414748

[B142] Lawson E. A. Olszewski P. K. Weller A. Blevins J. E. (2020). The role of oxytocin in regulation of appetitive behaviour, body weight and glucose homeostasis. *J. Neuroendocrinol.* 32:e12805. 10.1111/jne.12805 31657509 PMC7186135

[B143] Lee E. S. Uhm K. O. Lee Y. M. Kwon J. Park S. H. Soo K. H. (2008). Oxytocin stimulates glucose uptake in skeletal muscle cells through the calcium-CaMKK-AMPK pathway. *Regul. Pept.* 151 71–74. 10.1016/j.regpep.2008.05.001 18555543

[B144] Lee M. R. Scheidweiler K. B. Diao X. X. Akhlaghi F. Cummins A. Huestis M. A. (2018). Oxytocin by intranasal and intravenous routes reaches the cerebrospinal fluid in rhesus macaques: determination using a novel oxytocin assay. *Mol. Psychiatry* 23 115–122. 10.1038/mp.2017.27 28289281 PMC5862033

[B145] Lein E. S. Hawrylycz M. J. Ao N. Ayres M. Bensinger A. Bernard A. (2007). Genome-wide atlas of gene expression in the adult mouse brain. *Nature* 445 168–176. 17151600 10.1038/nature05453

[B146] Leitner C. Bartness T. J. (2009). Acute brown adipose tissue temperature response to cold in monosodium glutamate-treated Siberian hamsters. *Brain Res.* 1292 38–51. 10.1016/j.brainres.2009.07.062 19643091 PMC3995981

[B147] Leng G. Ludwig M. (2008). Neurotransmitters and peptides: whispered secrets and public announcements. *J. Physiol.* 586 5625–5632. 10.1113/jphysiol.2008.159103 18845614 PMC2655398

[B148] Leng G. Ludwig M. (2016a). Intranasal oxytocin: myths and delusions. *Biol. Psychiatry* 79 243–250. 10.1016/j.biopsych.2015.05.003 26049207

[B149] Leng G. Ludwig M. (2016b). Reply to: improving research standards to restore trust in intranasal oxytocin. *Biol. Psychiatry* 79 e55–e56. 10.1016/j.biopsych.2015.08.030 26435221

[B150] Leng G. Ludwig M. (2016c). Reply to: intranasal oxytocin mechanisms can be better understood, but its effects on social cognition and behavior are not to be sniffed at. *Biol. Psychiatry* 79 e51–e52. 10.1016/j.biopsych.2015.06.022 26212899

[B151] Leng G. Onaka T. Caquineau C. Sabatier N. Tobin V. A. Takayanagi Y. (2008). Oxytocin and appetite. *Prog. Brain Res.* 170 137–151. 18655879 10.1016/S0079-6123(08)00413-5

[B152] Leng G. Sabatier N. (2017). Oxytocin - the sweet hormone? *Trends Endocrinol. Metab. TEM* 28 365–376. 28283319 10.1016/j.tem.2017.02.007

[B153] Li L. Kong X. Liu H. Liu C. (2007). Systemic oxytocin and vasopressin excite gastrointestinal motility through oxytocin receptor in rabbits. *Neurogastroenterol. Motil.* 19 839–844. 10.1111/j.1365-2982.2007.00953.x 17883435

[B154] Liao P. Y. Chiu Y. M. Yu J. H. Chen S. K. (2020). Mapping central projection of oxytocin neurons in unmated mice using cre and alkaline phosphatase reporter. *Front. Neuroanat.* 14:559402. 10.3389/fnana.2020.559402 33192340 PMC7604466

[B155] Liu C. M. Hsu T. M. Suarez A. N. Subramanian K. S. Fatemi R. A. Cortella A. M. (2020b). Central oxytocin signaling inhibits food reward-motivated behaviors and VTA dopamine responses to food-predictive cues in male rats. *Hormon. Behav.* 126:104855. 10.1016/j.yhbeh.2020.104855 32991888 PMC7757852

[B156] Liu C. M. Davis E. A. Suarez A. N. Wood R. I. Noble E. E. Kanoski S. E. (2020a). Sex differences and estrous influences on oxytocin control of food intake. *Neuroscience* 447 63–73. 31738883 10.1016/j.neuroscience.2019.10.020 PMC7225050

[B157] Lokrantz C. M. vnas-Moberg K. U. Kaplan J. M. (1997). Effects of central oxytocin administration on intraoral intake of glucose in deprived and nondeprived rats. *Physiol. Behav.* 62 347–352. 10.1016/s0031-9384(97)00021-8 9251978

[B158] Loup F. Tribollet E. Dubois-Dauphin M. Dreifuss J. J. (1991). Localization of high-affinity binding sites for oxytocin and vasopressin in the human brain. An autoradiographic study. *Brain Res.* 555 220–232. 10.1016/0006-8993(91)90345-v 1657300

[B159] Loup F. Tribollet E. Dubois-Dauphin M. Pizzolato G. Dreifuss J. J. (1989). Localization of oxytocin binding sites in the human brainstem and upper spinal cord: an autoradiographic study. *Brain Res.* 500 223–230. 2557960 10.1016/0006-8993(89)90317-x

[B160] Ludwig M. Leng G. (2006). Dendritic peptide release and peptide-dependent behaviours. *Nat. Rev. Neurosci.* 7 126–136. 10.1038/nrn1845 16429122

[B161] Ludwig M. Tobin V. A. Callahan M. F. Papadaki E. Becker A. Engelmann M. (2013). Intranasal application of vasopressin fails to elicit changes in brain immediate early gene expression, neural activity and behavioural performance of rats. *J. Neuroendocrinol.* 25 655–667. 10.1111/jne.12046 23656518 PMC3697072

[B162] Luo F. Mu Y. Gao C. Xiao Y. Zhou Q. Yang Y. (2019). Whole-brain patterns of the presynaptic inputs and axonal projections of BDNF neurons in the paraventricular nucleus. *J. Genet. Genom.* 46 31–40. 10.1016/j.jgg.2018.11.004 30745213

[B163] MacDonald E. Dadds M. R. Brennan J. L. Williams K. Levy F. Cauchi A. J. (2011). A review of safety, side-effects and subjective reactions to intranasal oxytocin in human research. *Psychoneuroendocrinology* 36 1114–1126. 10.1016/j.psyneuen.2011.02.015 21429671

[B164] Maejima Y. Aoyama M. Sakamoto K. Jojima T. Aso Y. Takasu K. (2017). Impact of sex, fat distribution and initial body weight on oxytocin’s body weight regulation. *Sci. Rep.* 7:8599. 10.1038/s41598-017-09318-7 28819236 PMC5561196

[B165] Maejima Y. Horita S. Otsuka A. Hidema S. Nishimori K. Shimomura K. (2020). Oral oxytocin delivery with proton pump inhibitor pretreatment decreases food intake. *Peptides* 128:170312. 10.1016/j.peptides.2020.170312 32298773

[B166] Maejima Y. Iwasaki Y. Yamahara Y. Kodaira M. Sedbazar U. Yada T. (2011). Peripheral oxytocin treatment ameliorates obesity by reducing food intake and visceral fat mass. *Aging* 3 1169–1177. 10.18632/aging.100408 22184277 PMC3273897

[B167] Maejima Y. Kato S. Horita S. Ueta Y. Takenoshita S. Kobayashi K. (2019). The hypothalamus to brainstem circuit suppresses late-onset body weight gain. *Sci. Rep.* 9:18360. 10.1038/s41598-019-54870-z 31798010 PMC6892811

[B168] Maejima Y. Rita R. S. Santoso P. Aoyama M. Hiraoka Y. Nishimori K. (2015). Nasal oxytocin administration reduces food intake without affecting locomotor activity and glycemia with c-fos induction in limited brain areas. *Neuroendocrinology* 101 35–44. 10.1159/000371636 25573626

[B169] Maejima Y. Sakuma K. Santoso P. Gantulga D. Katsurada K. Ueta Y. (2014). Oxytocinergic circuit from paraventricular and supraoptic nuclei to arcuate POMC neurons in hypothalamus. *FEBS Lett.* 588 4404–4412. 10.1016/j.febslet.2014.10.010 25448678

[B170] Maejima Y. Sedbazar U. Suyama S. Kohno D. Onaka T. Takano E. (2009). Nesfatin-1-regulated oxytocinergic signaling in the paraventricular nucleus causes anorexia through a leptin-independent melanocortin pathway. *Cell Metab.* 10 355–365. 10.1016/j.cmet.2009.09.002 19883614

[B171] Matarazzo V. Schaller F. Nedelec E. Benani A. Penicaud L. Muscatelli F. (2012). Inactivation of Socs3 in the hypothalamus enhances the hindbrain response to endogenous satiety signals via oxytocin signaling. *J. Neurosci.* 32 17097–17107. 10.1523/JNEUROSCI.1669-12.2012 23197703 PMC6621871

[B172] McCann M. J. Verbalis J. G. Stricker E. M. (1989). LiCl and CCK inhibit gastric emptying and feeding and stimulate OT secretion in rats. *Am. J. Physiol.* 256 R463–R468. 10.1152/ajpregu.1989.256.2.R463 2537039

[B173] McCormack S. E. Blevins J. E. Lawson E. A. (2020). Metabolic effects of oxytocin. *Endocrine Rev.* 41 121–145. 10.1210/endrev/bnz012 PMC7012298 31803919

[B174] Mens W. B. Witter A. van Wimersma Greidanus T. B. (1983). Penetration of neurohypophyseal hormones from plasma into cerebrospinal fluid (CSF): half-times of disappearance of these neuropeptides from CSF. *Brain Res.* 262 143–149. 10.1016/0006-8993(83)90478-x 6831225

[B175] Meredith M. E. Salameh T. S. Banks W. A. (2015). Intranasal delivery of proteins and peptides in the treatment of neurodegenerative diseases. *AAPS J.* 17 780–787. 25801717 10.1208/s12248-015-9719-7 PMC4476983

[B176] Michalakis K. Ilias I. (2020). SARS-CoV-2 infection and obesity: common inflammatory and metabolic aspects. *Diabetes Metab. Syndr.* 14 469–471. 32387864 10.1016/j.dsx.2020.04.033 PMC7189186

[B177] Miller G. (2013). The promise and perils of oxytocin. *Science* 339 267–269. 10.1126/science.339.6117.267 23329028

[B178] Modi M. E. Connor-Stroud F. Landgraf R. Young L. J. Parr L. A. (2014). Aerosolized oxytocin increases cerebrospinal fluid oxytocin in rhesus macaques. *Psychoneuroendocrinology* 45 49–57. 10.1016/j.psyneuen.2014.02.011 24845176 PMC4120060

[B179] Monstein H. J. Grahn N. Truedsson M. Ohlsson B. (2004). Oxytocin and oxytocin-receptor mRNA expression in the human gastrointestinal tract: a polymerase chain reaction study. *Regul. Pept.* 119 39–44. 15093695 10.1016/j.regpep.2003.12.017

[B180] Montag C. Brockmann E. M. Bayerl M. Rujescu D. Muller D. J. Gallinat J. (2012). Oxytocin and oxytocin receptor gene polymorphisms and risk for schizophrenia: a case-control study. *World J. Biol. Psychiatry* 14 500–508. 22651577 10.3109/15622975.2012.677547

[B181] Monte O. Dal Noble P. L. Turchi J. Cummins A. Averbeck B. B. (2014). CSF and blood oxytocin concentration changes following intranasal delivery in macaque. *PLoS one* 9:e103677. 10.1371/journal.pone.0103677 25133536 PMC4136720

[B182] Moos F. Richard P. (1989). Paraventricular and supraoptic bursting oxytocin cells in rat are locally regulated by oxytocin and functionally related. *J. Physiol.* 408 1–18. 10.1113/jphysiol.1989.sp017442 2778722 PMC1190386

[B183] Morrison S. F. Madden C. J. Tupone D. (2014). Central neural regulation of brown adipose tissue thermogenesis and energy expenditure. *Cell Metab.* 19 741–756. 24630813 10.1016/j.cmet.2014.02.007 PMC4016184

[B184] Morton G. J. Thatcher B. S. Reidelberger R. D. Ogimoto K. Wolden-Hanson T. Baskin D. G. (2012). Peripheral oxytocin suppresses food intake and causes weight loss in diet-induced obese rats. *Am. J. Physiol. Endoc. M.* 302 E134–E144. 10.1152/ajpendo.00296.2011 22008455 PMC3328087

[B185] Muchmore D. B. Little S. A. de Haen C. dual A. (1981). mechanism of action of ocytocin in rat epididymal fat cells. *J. Biol. Chem.* 256 365–372. 7005215

[B186] Mullis K. Kay K. Williams D. L. (2013). Oxytocin action in the ventral tegmental area affects sucrose intake. *Brain Res.* 1513 85–91. 10.1016/j.brainres.2013.03.026 23548602 PMC3739708

[B187] Nasanbuyan N. Yoshida M. Takayanagi Y. Inutsuka A. Nishimori K. Yamanaka A. (2018). Oxytocin-oxytocin receptor systems facilitate social defeat posture in male mice. *Endocrinology* 159 763–775. 10.1210/en.2017-00606 29186377

[B188] Nation D. A. Szeto A. Mendez A. J. Brooks L. G. Zaias J. Herderick E. E. (2010). Oxytocin attenuates atherosclerosis and adipose tissue inflammation in socially isolated ApoE-/- mice. *Psychos. Med.* 72 376–382. 10.1097/PSY.0b013e3181d74c48 20368478 PMC4784697

[B189] Nedergaard J. Cannon B. (2014). The browning of white adipose tissue: some burning issues. *Cell Metab.* 20 396–407. 25127354 10.1016/j.cmet.2014.07.005

[B190] Neumann I. D. Maloumby R. Beiderbeck D. I. Lukas M. Landgraf R. (2013). Increased brain and plasma oxytocin after nasal and peripheral administration in rats and mice. *Psychoneuroendocrinology* 38 1985–1993. 10.1016/j.psyneuen.2013.03.003 23579082

[B191] Nguyen N. T. Magno C. P. Lane K. T. Hinojosa M. W. Lane J. S. (2008). Association of hypertension, diabetes, dyslipidemia, and metabolic syndrome with obesity: findings from the National Health and Nutrition Examination Survey, 1999 to 2004. *J. Am. College Surg.* 207 928–934. 10.1016/j.jamcollsurg.2008.08.022 19183541

[B192] Noble E. E. Billington C. J. Kotz C. M. Wang C. (2014). Oxytocin in the ventromedial hypothalamic nucleus reduces feeding and acutely increases energy expenditure. *Am. J. Physiol. Regul. Integr. Comp. Physiol.* 307 R737–R745. 10.1152/ajpregu.00118.2014 24990860 PMC4166752

[B193] Nunn N. Womack M. Dart C. Barrett-Jolley R. (2011). Function and pharmacology of spinally-projecting sympathetic pre-autonomic neurones in the paraventricular nucleus of the hypothalamus. *Curr. Neuropharmacol.* 9 262–277. 10.2174/157015911795596531 22131936 PMC3131718

[B194] Ogden C. L. Carroll M. D. Kit B. K. Flegal K. M. (2012). Prevalence of obesity in the United States, 2009-2010. *NCHS Data Brief* 82 1–8. 22617494

[B195] Ohlsson B. Truedsson M. Djerf P. Sundler F. (2006). Oxytocin is expressed throughout the human gastrointestinal tract. *Regul. Pept.* 135 7–11. 16678285 10.1016/j.regpep.2006.03.008

[B196] Oldfield B. J. Giles M. E. Watson A. Anderson C. Colvill L. M. McKinley M. J. (2002). The neurochemical characterisation of hypothalamic pathways projecting polysynaptically to brown adipose tissue in the rat. *Neuroscience* 110 515–526. 10.1016/s0306-4522(01)00555-3 11906790

[B197] Olson B. R. Drutarosky M. D. Stricker E. M. Verbalis J. G. (1991b). Brain oxytocin receptor antagonism blunts the effects of anorexigenic treatments in rats: evidence for central oxytocin inhibition of food intake. *Endocrinology* 129 785–791. 10.1210/endo-129-2-785 1649746

[B198] Olson B. R. Drutarosky M. D. Chow M. S. Hruby V. J. Stricker E. M. Verbalis J. G. (1991a). Oxytocin and an oxytocin agonist administered centrally decrease food intake in rats. *Peptides* 1991 113–118. 10.1016/0196-9781(91)90176-p 1646995

[B199] Olson B. R. Hoffman G. E. Sved A. F. Stricker E. M. Verbalis J. G. (1992). Cholecystokinin induces c-fos expression in hypothalamic oxytocinergic neurons projecting to the dorsal vagal complex. *Brain Res.* 569 238–248. 10.1016/0006-8993(92)90635-m 1371708

[B200] Olszewski P. K. Klockars A. Olszewska A. M. Fredriksson R. Schioth H. B. Levine A. S. (2010). Molecular, immunohistochemical, and pharmacological evidence of oxytocin’s role as inhibitor of carbohydrate but not fat intake. *Endocrinology* 151 4736–4744. 10.1210/en.2010-0151 20685878 PMC2946140

[B201] Olszewski P. K. Wirth M. M. Shaw T. J. Grace M. K. Billington C. J. Giraudo S. Q. (2001). Role of alpha-MSH in the regulation of consummatory behavior: immunohistochemical evidence. *Am. J. Physiol. Regul. Integr. Comp. Physiol.* 281 R673–R680. 10.1152/ajpregu.2001.281.2.R673 11448874

[B202] Ong Z. Y. Alhadeff A. L. Grill H. J. (2015). Medial nucleus tractus solitarius oxytocin receptor signaling and food intake control: the role of gastrointestinal satiation signal processing. *Am. J. Physiol. Regul. Integr. Comp. Physiol.* 308 R800–R806. 10.1152/ajpregu.00534.2014 25740340 PMC4421744

[B203] Ong Z. Y. Bongiorno D. M. Hernando M. A. Grill H. J. (2017). Effects of endogenous oxytocin receptor signaling in nucleus tractus solitarius on satiation-mediated feeding and thermogenic control in male rats. *Endocrinology* 158 2826–2836. 10.1210/en.2017-00200 28575174 PMC5659667

[B204] Ott V. Finlayson G. Lehnert H. Heitmann B. Heinrichs M. Born J. (2013). Oxytocin reduces reward-driven food intake in humans. *Diabetes* 62 3418–3425. 23835346 10.2337/db13-0663 PMC3781467

[B205] Paiva L. Lozic M. Allchorne A. Grinevich V. Ludwig M. (2021). Identification of peripheral oxytocin-expressing cells using systemically applied cell-type specific adeno-associated viral vector. *J. Neuroendocrinol.* 33:e12970. 10.1111/jne.12970 33851744

[B206] Paulin C. Dubois P. M. Czernichow P. Dubois M. P. (1978). Immunocytological evidence for oxytocin neurons in the human fetal hypothalamus. *Cell Tissue Res.* 188 259–264. 10.1007/BF00222635 348325

[B207] Peris J. MacFadyen K. Smith J. A. de Kloet A. D. Wang L. Krause E. G. (2016). Oxytocin receptors are expressed on dopamine and glutamate neurons in the mouse ventral tegmental area that project to nucleus accumbens and other mesolimbic targets. *J. Comp. Neurol.* 525 1094–1108. 10.1002/cne.24116 27615433 PMC6483090

[B208] Peris J. MacFadyen K. Smith J. A. de Kloet A. D. Wang L. Krause E. G. (2017). Oxytocin receptors are expressed on dopamine and glutamate neurons in the mouse ventral tegmental area that project to nucleus accumbens and other mesolimbic targets. *J. Comp. Neurol.* 525 1094–1108. 27615433 10.1002/cne.24116 PMC6483090

[B209] Peters S. Slattery D. A. Uschold-Schmidt N. Reber S. O. Neumann I. D. (2014). Dose-dependent effects of chronic central infusion of oxytocin on anxiety, oxytocin receptor binding and stress-related parameters in mice. *Psychoneuroendocrinology* 42 225–236. 10.1016/j.psyneuen.2014.01.021 24636519

[B210] Petersson M. (2002). Cardiovascular effects of oxytocin. *Prog. Brain Res.* 139 281–288. 12436943 10.1016/s0079-6123(02)39024-1

[B211] Petersson M. Alster P. Lundeberg T. vnas-Moberg K. U. (1996). Oxytocin causes a long-term decrease of blood pressure in female and male rats. *Physiol. Behav.* 60 1311–1315. 8916187 10.1016/s0031-9384(96)00261-2

[B212] Pinder A. J. Dresner M. Calow C. Shorten G. D. O’Riordan J. Johnson R. (2002). Haemodynamic changes caused by oxytocin during caesarean section under spinal anaesthesia. *Int. J. Obstet. Anesth.* 11 156–159. 15321540 10.1054/ijoa.2002.0970

[B213] Plante E. Menaouar A. Danalache B. A. Yip D. Broderick T. L. Chiasson J. L. (2015). Oxytocin treatment prevents the cardiomyopathy observed in obese diabetic male db/db mice. *Endocrinology* 156 1416–1428. 10.1210/en.2014-1718 25562615

[B214] Plessow F. Marengi D. A. Perry S. K. Felicione J. M. Franklin R. Holmes T. M. (2018). Effects of intranasal oxytocin on the blood oxygenation level-dependent signal in food motivation and cognitive control pathways in overweight and obese men. *Neuropsychopharmacology* 43 638–645. 10.1038/npp.2017.226 28930284 PMC5770767

[B215] Pow D. V. Morris J. F. (1989). Dendrites of hypothalamic magnocellular neurons release neurohypophysial peptides by exocytosis. *Neuroscience* 32 435–439. 2586758 10.1016/0306-4522(89)90091-2

[B216] Qin J. Feng M. Wang C. Ye Y. Wang P. S. Liu C. (2009). Oxytocin receptor expressed on the smooth muscle mediates the excitatory effect of oxytocin on gastric motility in rats. *Neurogastroenterol. Motil.* 21 430–438. 10.1111/j.1365-2982.2009.01282.x 19309416

[B217] Ragen B. J. Bales K. L. (2013). “Oxytocin and vasopressin in non-human primates,” in *Oxytocin, Vasopressin and Related Peptides in the Regulation of Behavior*, eds Choleris E. Pfaff D. W. Kavaliers M. (Cambridge: Cambridge University Press), 288–306.

[B218] Rajagopal S. Cheskin L. J. (2021). In overweight or obese adults without diabetes, semaglutide increased weight loss and GI disorders. *Ann. Intern. Med.* 174:JC80. 10.7326/ACPJ202107200-080 34224258

[B219] Rault J. L. Carter C. S. Garner J. P. Marchant-Forde J. N. Richert B. T. Lay D. C. Jr. (2013). Repeated intranasal oxytocin administration in early life dysregulates the HPA axis and alters social behavior. *Physiol. Behav.* 112-113 40–48. 10.1016/j.physbeh.2013.02.007 23481917

[B220] Reiter M. K. Kremarik P. Freund-Mercier M. J. Stoeckel M. E. Desaulles E. Feltz P. (1994). Localization of oxytocin binding sites in the thoracic and upper lumbar spinal cord of the adult and postnatal rat: a histoautoradiographic study. *Eur. J. Neurosci.* 6 98–104. 10.1111/j.1460-9568.1994.tb00251.x 8130936

[B221] Renaud L. P. Bourque C. W. (1991). Neurophysiology and neuropharmacology of hypothalamic magnocellular neurons secreting vasopressin and oxytocin. *Prog. Neurobiol.* 36 131–169. 10.1016/0301-0082(91)90020-2 1998074

[B222] Rinaman L. (1998). Oxytocinergic inputs to the nucleus of the solitary tract and dorsal motor nucleus of the vagus in neonatal rats. *J. Comp. Neurol.* 399 101–109. 10.1002/(sici)1096-9861(19980914)399:1&lt;101::aid-cne8&gt;3.0.co;2-5 9725704

[B223] Rinaman L. Rothe E. E. (2002). GLP-1 receptor signaling contributes to anorexigenic effect of centrally administered oxytocin in rats. *Am. J. Physiol. Regul. Integr. Comp. Physiol.* 283 R99–R106. 10.1152/ajpregu.00008.2002 12069935

[B224] Ring R. H. Malberg J. E. Potestio L. Ping J. Boikess S. Luo B. (2006). Anxiolytic-like activity of oxytocin in male mice: behavioral and autonomic evidence, therapeutic implications. *Psychopharmacology* 185 218–225. 10.1007/s00213-005-0293-z 16418825

[B225] Ring R. H. Schechter L. E. Leonard S. K. Dwyer J. M. Platt B. J. Graf R. (2010). Receptor and behavioral pharmacology of WAY-267464, a non-peptide oxytocin receptor agonist. *Neuropharmacology* 58 69–77. 19615387 10.1016/j.neuropharm.2009.07.016

[B226] Roberts Z. S. Wolden-Hanson T. H. Matsen M. E. Ryu V. Vaughan C. H. Graham J. L. (2017). Chronic hindbrain administration of oxytocin is sufficient to elicit weight loss in diet-induced obese rats. *Am. J. Physiol. Regul. Integr. Comp. Physiol.* 313 R357–R371. 10.1152/ajpregu.00169.2017 28747407 PMC5668612

[B227] Rodgers R. J. Tschop M. H. Wilding J. P. (2012). Anti-obesity drugs: past, present and future. *Dis. Models Mech.* 5 621–626. 10.1242/dmm.009621 PMC3424459 22915024

[B228] Rogers R. C. Hermann G. E. (1987). Oxytocin, oxytocin antagonist, TRH, and hypothalamic paraventricular nucleus stimulation effects on gastric motility. *Peptides* 8 505–513. 10.1016/0196-9781(87)90017-9 3116510

[B229] Romero-Fernandez W. Borroto-Escuela D. O. Agnati L. F. Fuxe K. (2013). Evidence for the existence of dopamine D2-oxytocin receptor heteromers in the ventral and dorsal striatum with facilitatory receptor-receptor interactions. *Mol. Psychiatry* 18 849–850. 10.1038/mp.2012.103 22824810

[B230] Rosen G. J. de Vries G. J. Goldman S. L. Goldman B. D. Forger N. G. (2008). Distribution of oxytocin in the brain of a eusocial rodent. *Neuroscience* 155 809–817. 18582538 10.1016/j.neuroscience.2008.05.039 PMC2614305

[B231] Rosenbaum M. Goldsmith R. Bloomfield D. Magnano A. Weimer L. Heymsfield S. (2005). Low-dose leptin reverses skeletal muscle, autonomic, and neuroendocrine adaptations to maintenance of reduced weight. *J. Clin. Investig.* 115 3579–3586. 10.1172/JCI25977 16322796 PMC1297250

[B232] Rosenbaum M. Kissileff H. R. Mayer L. E. Hirsch J. Leibel R. L. (2010). Energy intake in weight-reduced humans. *Brain Res.* 1350 95–102. 20595050 10.1016/j.brainres.2010.05.062 PMC2926239

[B233] Rosenbaum M. Murphy E. M. Heymsfield S. B. Matthews D. E. Leibel R. L. (2002). Low dose leptin administration reverses effects of sustained weight-reduction on energy expenditure and circulating concentrations of thyroid hormones. *J. Clin. Endocrinol. Metab.* 87 2391–2394. 10.1210/jcem.87.5.8628 11994393

[B234] Ross H. E. Cole C. D. Smith Y. Neumann I. D. Landgraf R. Murphy A. Z. (2009). Characterization of the oxytocin system regulating affiliative behavior in female prairie voles. *Neuroscience* 162 892–903. 19482070 10.1016/j.neuroscience.2009.05.055 PMC2744157

[B235] Ryan P. J. Ross S. I. Campos C. A. Derkach V. A. Palmiter R. D. (2017). Oxytocin-receptor-expressing neurons in the parabrachial nucleus regulate fluid intake. *Nat. Neurosci.* 20 1722–1733. 10.1038/s41593-017-0014-z 29184212 PMC5705772

[B236] Sabatier N. Leng G. Menzies J. (2013). Oxytocin, feeding, and satiety. *Front. Endocrinol.* 4:35. 10.3389/fendo.2013.00035 23518828 PMC3603288

[B237] Sabatier N. Rowe I. Leng G. (2007). Central release of oxytocin and the ventromedial hypothalamus. *Biochem. Soc. Trans.* 35 1247–1251. 17956323 10.1042/BST0351247

[B238] Sawchenko P. E. Swanson L. W. (1982). Immunohistochemical identification of neurons in the paraventricular nucleus of the hypothalamus that project to the medulla or to the spinal cord in the rat. *J. Comp. Neurol.* 205 260–272. 6122696 10.1002/cne.902050306

[B239] Sawchenko P. E. Swanson L. W. Vale W. W. (1984). Corticotropin-releasing factor: co-expression within distinct subsets of oxytocin-, vasopressin-, and neurotensin-immunoreactive neurons in the hypothalamus of the male rat. *J. Neurosci.* 4 1118–1129. 10.1523/JNEUROSCI.04-04-01118.1984 6609226 PMC6564788

[B240] Schaffler N. Binart Scholmerich J. Buchler C. (2005). Hypothesis paper Brain talks with fat–evidence for a hypothalamic-pituitary-adipose axis? *Neuropeptides* 39 363–367. 10.1016/j.npep.2005.06.003 16040119

[B241] Schellekens H. Dinan T. G. Cryan J. F. (2013). Taking two to tango: a role for ghrelin receptor heterodimerization in stress and reward. *Front. Neurosci.* 7:148. 10.3389/fnins.2013.00148 24009547 PMC3757321

[B242] Schorr M. Marengi D. A. Pulumo R. L. Yu E. Eddy K. T. Klibanski A. (2017). Oxytocin and its relationship to body composition, bone mineral density, and hip geometry across the weight spectrum. *J. Clin. Endocrinol. Metab.* 102 2814–2824. 10.1210/jc.2016-3963 28586943 PMC5546854

[B243] Schorscher-Petcu A. Dupre A. Tribollet E. (2009). Distribution of vasopressin and oxytocin binding sites in the brain and upper spinal cord of the common marmoset. *Neurosci. Lett.* 461 217–222. 10.1016/j.neulet.2009.06.016 19539696

[B244] Schumacher M. Coirini H. Frankfurt M. McEwen B. S. (1989). Localized actions of progesterone in hypothalamus involve oxytocin. *Proc. Natl. Acad. Sci. U.S.A.* 86 6798–6801. 10.1073/pnas.86.17.6798 2549547 PMC297933

[B245] Schwartz A. Doucet E. (2010). Relative changes in resting energy expenditure during weight loss: a systematic review. *Obes. Rev.* 11 531–547. 10.1111/j.1467-789X.2009.00654.x 19761507

[B246] Schwartz M. W. Woods S. C. Porte D. Jr. Seeley R. J. Baskin D. G. (2000). Central nervous system control of food intake. *Nature* 404 661–671. 10766253 10.1038/35007534

[B247] Seeley R. J. Yagaloff K. A. Fisher S. L. Burn P. Thiele T. E. van Dijk G. (1997). Melanocortin receptors in leptin effects. *Nature* 390:349. 10.1038/37016 9389472

[B248] Seelke A. M. Rhine M. A. Khun K. Shweyk A. N. Scott A. M. Bond J. M. (2018). Intranasal oxytocin reduces weight gain in diet-induced obese prairie voles. *Physiol. Behav.* 196 67–77. 10.1016/j.physbeh.2018.08.007 30144467 PMC6195438

[B249] Shahrokh D. K. Zhang T. Y. Diorio J. Gratton A. Meaney M. J. (2010). Oxytocin-dopamine interactions mediate variations in maternal behavior in the rat. *Endocrinology* 151 2276–2286. 10.1210/en.2009-1271 20228171 PMC2869254

[B250] Shi H. Bartness T. J. (2001). Neurochemical phenotype of sympathetic nervous system outflow from brain to white fat. *Brain Res. Bull.* 54 375–385. 10.1016/s0361-9230(00)00455-x 11306188

[B251] Smith A. S. Korgan A. C. Young W. S. (2019). Oxytocin delivered nasally or intraperitoneally reaches the brain and plasma of normal and oxytocin knockout mice. *Pharmacol. Res.* 146:104324. 10.1016/j.phrs.2019.104324 PMC6679720 31238093

[B252] Smyth S. Heron A. (2006). Diabetes and obesity: the twin epidemics. *Nat. Med.* 12 75–80. 16397575 10.1038/nm0106-75

[B253] Snider B. Geiser A. Yu X. P. Beebe E. C. Willency J. A. Qing K. (2019). Long-acting and selective oxytocin peptide analogs show antidiabetic and antiobesity effects in male mice. *J. Endocrine Soc.* 3 1423–1444. 10.1210/js.2019-00004 31286109 PMC6608564

[B254] Sofroniew M. V. (1983). Morphology of vasopressin and oxytocin neurones and their central and vascular projections. *Prog. Brain Res.* 60 101–114. 6198686 10.1016/S0079-6123(08)64378-2

[B255] Sofroniew M. V. Weindl A. Schrell U. Wetzstein R. (1981). Immunohistochemistry of vasopressin, oxytocin and neurophysin in the hypothalamus and extrahypothalamic regions of the human and primate brain. *Acta Histochem. Suppl.* 24 79–95. 6785843

[B256] Song C. K. Vaughan C. H. Keen-Rhinehart E. Harris R. B. Richard D. Bartness T. J. (2008). Melanocortin-4 receptor mRNA expressed in sympathetic outflow neurons to brown adipose tissue: neuroanatomical and functional evidence. *Am. J. Physiol. Regul. Integr. Comp. Physiol.* 295 R417–R428. 10.1152/ajpregu.00174.2008 18550869 PMC2519921

[B257] Stanley S. Pinto S. Segal J. Perez C. A. Viale A. DeFalco J. (2010). Identification of neuronal subpopulations that project from hypothalamus to both liver and adipose tissue polysynaptically. *Proc. Natl. Acad. Sci. U.S.A.* 107 7024–7029. 10.1073/pnas.1002790107 20351287 PMC2872469

[B258] Striepens N. Kendrick K. M. Hanking V. Landgraf R. Wullner U. Maier W. (2013). Elevated cerebrospinal fluid and blood concentrations of oxytocin following its intranasal administration in humans. *Sci. Rep.* 3:3440. 10.1038/srep03440 PMC3853684 24310737

[B259] Striepens N. Kendrick K. M. Maier W. Hurlemann R. (2011). Prosocial effects of oxytocin and clinical evidence for its therapeutic potential. *Front. Neuroendocrinol.* 32 426–450. 10.1016/j.yfrne.2011.07.001 21802441

[B260] Striepens N. Schroter F. Stoffel-Wagner B. Maier W. Hurlemann R. Scheele D. (2016). Oxytocin enhances cognitive control of food craving in women. *Hum. Brain Mapp.* 37 4276–4285. 10.1002/hbm.23308 27381253 PMC6867465

[B261] Sukhov R. R. Walker L. C. Rance N. E. Price D. L. Young W. S. III (1993). Vasopressin and oxytocin gene expression in the human hypothalamus. *J. Comp. Neurol.* 337 295–306. 8277003 10.1002/cne.903370210 PMC9883978

[B262] Sun L. Lizneva D. Ji Y. Colaianni G. Hadelia E. Gumerova A. (2019). Oxytocin regulates body composition. *Proc. Natl. Acad. Sci. U.S.A.* 116 26808–26815. 10.1073/pnas.1913611116 PMC6936484 31843930

[B263] Sutton A. K. Pei H. Burnett K. H. Myers M. G. Jr. Rhodes C. J. Olson D. P. (2014). Control of food intake and energy expenditure by Nos1 neurons of the paraventricular hypothalamus. *J. Neurosci.* 34 15306–15318. 25392498 10.1523/JNEUROSCI.0226-14.2014 PMC4228133

[B264] Swaab D. F. Nijveldt F. Pool C. W. (1975a). Distribution of oxytocin and vasopressin in the rat supraoptic and paraventricular nucleus. *J. Endocrinol.* 67 461–462. 1206330 10.1677/joe.0.0670461

[B265] Swaab D. F. Pool C. W. Nijveldt F. (1975b). Immunofluorescence of vasopressin and oxytocin in the rat hypothalamo-neurohypophypopseal system. *J. Neural Transm.* 36 195–215. 10.1007/BF01253126 1100784

[B266] Szabo R. Menesi R. Szalai Z. Daruka L. Toth G. Gardi J. (2019). New metabolic influencer on oxytocin release: the ghrelin. *Molecules* 24:735. 10.3390/molecules24040735 30781678 PMC6413225

[B267] Tachibana M. Kagitani-Shimono K. Mohri I. Yamamoto T. Sanefuji W. Nakamura A. (2013). Long-term administration of intranasal oxytocin is a safe and promising therapy for early adolescent boys with autism spectrum disorders. *J. Child Adolesc. Psychopharmacol.* 23 123–127. 10.1089/cap.2012.0048 23480321

[B268] Takayanagi Y. Kasahara Y. Onaka T. Takahashi N. Kawada T. Nishimori K. (2008). Oxytocin receptor-deficient mice developed late-onset obesity. *Neuroreport* 19 951–955. 18520999 10.1097/WNR.0b013e3283021ca9

[B269] Tamma R. Colaianni G. Zhu L. L. DiBenedetto A. Greco G. Montemurro G. (2009). Oxytocin is an anabolic bone hormone. *Proc. Natl. Acad. Sci. U.S.A.* 106 7149–7154. 19369205 10.1073/pnas.0901890106 PMC2678458

[B270] Targher G. Mantovani A. Wang X. B. Yan H. D. Sun Q. F. Pan K. H. (2020). Patients with diabetes are at higher risk for severe illness from COVID-19. *Diabetes Metab.* 46 335–337. 32416321 10.1016/j.diabet.2020.05.001 PMC7255326

[B271] Terrillon S. Durroux T. Mouillac B. Breit A. Ayoub M. A. Taulan M. (2003). Oxytocin and vasopressin V1a and V2 receptors form constitutive homo- and heterodimers during biosynthesis. *Mol. Endocrinol.* 17 677–691. 10.1210/me.2002-0222 12554793

[B272] Thienel M. Fritsche A. Heinrichs M. Peter A. Ewers M. Lehnert H. (2016). Oxytocin’s inhibitory effect on food intake is stronger in obese than normal-weight men. *Int. J. Obes.* 40 1707–1714. 10.1038/ijo.2016.149 27553712 PMC5116063

[B273] Tribollet E. Dubois-Dauphin M. Dreifuss J. J. Barberis C. Jard S. (1992). Oxytocin receptors in the central nervous system. Distribution, development, and species differences. *Ann. N.Y. Acad. Sci.* 652 29–38. 1320828 10.1111/j.1749-6632.1992.tb34343.x

[B274] Tsuda T. Ueno Y. Yoshikawa T. Kojo H. Osawa T. (2006). Microarray profiling of gene expression in human adipocytes in response to anthocyanins. *Biochem. Pharmacol.* 71 1184–1197. 10.1016/j.bcp.2005.12.042 16483547

[B275] Tung Y. C. Ma M. Piper S. Coll A. O’Rahilly S. Yeo G. S. (2008). Novel leptin-regulated genes revealed by transcriptional profiling of the hypothalamic paraventricular nucleus. *J. Neurosci.* 28 12419–12426. 10.1523/JNEUROSCI.3412-08.2008 19020034 PMC2650686

[B276] Vaccari C. Lolait S. J. Ostrowski N. L. (1998). Comparative distribution of vasopressin V1b and oxytocin receptor messenger ribonucleic acids in brain. *Endocrinology* 139 5015–5033. 10.1210/endo.139.12.6382 9832441

[B277] Vallera C. Choi L. O. Cha C. M. Hong R. W. (2017). Uterotonic medications: oxytocin, methylergonovine, carboprost, misoprostol. *Anesthesiol. Clin.* 35 207–219. 28526143 10.1016/j.anclin.2017.01.007

[B278] van Leeuwen F. W. van Heerikhuize J. van der Meulen G. Wolters P. (1985). Light microscopic autoradiographic localization of [3H]oxytocin binding sites in the rat brain, pituitary and mammary gland. *Brain Res.* 359 320–325. 10.1016/0006-8993(85)91443-x 4075153

[B279] van Zuiden M. Frijling J. L. Nawijn L. Koch S. B. J. Goslings J. C. Luitse J. S. (2017). Intranasal oxytocin to prevent posttraumatic stress disorder symptoms: a randomized controlled trial in emergency department patients. *Biol. Psychiatry* 81 1030–1040. 28087128 10.1016/j.biopsych.2016.11.012

[B280] Vaughan C. H. Shrestha Y. B. Bartness T. J. (2011). Characterization of a novel melanocortin receptor-containing node in the SNS outflow circuitry to brown adipose tissue involved in thermogenesis. *Brain Res.* 1411 17–27. 10.1016/j.brainres.2011.07.003 21802070 PMC3426614

[B281] Verbalis J. G. (1999). The brain oxytocin receptor(s)? *Front. Neuroendocrinol.* 20 146–156. 10328988 10.1006/frne.1999.0178

[B282] Verbalis J. G. Blackburn R. E. Hoffman G. E. Stricker E. M. (1995b). Establishing behavioral and physiological functions of central oxytocin: insights from studies of oxytocin and ingestive behaviors. *Adv. Exp. Med. Biol.* 395 209–225. 8713970

[B283] Verbalis J. G. Blackburn A. N. Hoffman G. E. Stricker E. M. (1995a). “Establishing behavioral and physiological functions of central oxytocin: insights from studies of oxytocin and ingestive behvaviors,” in *Oxytocin*, eds Ivell R. Russell J. (New York, NY: Plenum Press), 209–225. 8713970

[B284] Vila G. Riedl M. Resl M. van der Lely A. J. Hofland L. J. Clodi M. (2009). Systemic administration of oxytocin reduces basal and lipopolysaccharide-induced ghrelin levels in healthy men. *J. Endocrinol.* 203 175–179. 10.1677/JOE-09-0227 19587265

[B285] Wald H. S. Chandra A. Kalluri A. Ong Z. Y. Hayes M. R. Grill H. J. (2020). NTS and VTA oxytocin reduces food motivation and food seeking. *Am. J. Physiol. Regul. Integr. Comp. Physiol.* 319 R673–R683. 10.1152/ajpregu.00201.2020 33026822 PMC7792820

[B286] Wallace Fitzsimons S. E. Chruscicka B. Druelle C. Stamou P. Nally K. Dinan T. G. (2019). A ghrelin receptor and oxytocin receptor heterocomplex impairs oxytocin mediated signalling. *Neuropharmacology* 152 90–101. 10.1016/j.neuropharm.2018.12.022 30582955

[B287] Welch M. G. Margolis K. G. Li Z. Gershon M. D. (2014). Oxytocin regulates gastrointestinal motility, inflammation, macromolecular permeability, and mucosal maintenance in mice. *Am. J. Physiol. Gastrointest. Liver Physiol.* 307 G848–G862. 10.1152/ajpgi.00176.2014 25147234 PMC4200316

[B288] Welch M. G. Tamir H. Gross K. J. Chen J. Anwar M. Gershon M. D. (2009). Expression and developmental regulation of oxytocin (OT) and oxytocin receptors (OTR) in the enteric nervous system (ENS) and intestinal epithelium. *J. Comp. Neurol.* 512 256–270. 19003903 10.1002/cne.21872 PMC3097117

[B289] Wilding J. P. H. Batterham R. L. Calanna S. Davies M. Gaal L.F. Van Lingvay I. (2021). Once-weekly semaglutide in adults with overweight or obesity. *N. Engl. J. Med.* 384:989. 10.1056/NEJMoa2032183 33567185

[B290] Winter J. Meyer M. Berger I. Royer M. Bianchi M. Kuffner K. (2021). Chronic oxytocin-driven alternative splicing of Crfr2alpha induces anxiety. *Mol. Psychiatry* [Epub ahead of print]. 10.1038/s41380-021-01141-x 34035479 PMC10914602

[B291] Woods S. C. Schwartz M. W. Baskin D. G. Seeley R. J. (2000). Food intake and the regulation of body weight. *Annu. Rev. Psychol.* 51 255–277. 10751972 10.1146/annurev.psych.51.1.255

[B292] Wrobel L. Schorscher-Petcu A. Dupre A. Yoshida M. Nishimori K. Tribollet E. (2011). Distribution and identity of neurons expressing the oxytocin receptor in the mouse spinal cord. *Neurosci. Lett.* 495 49–54. 10.1016/j.neulet.2011.03.033 21419193

[B293] Wu C. L. Doong M. L. Wang P. S. (2008). Involvement of cholecystokinin receptor in the inhibition of gastrointestinal motility by oxytocin in ovariectomized rats. *Eur. J. Pharmacol.* 580 407–415. 10.1016/j.ejphar.2007.11.024 18078924

[B294] Wu C. L. Hung C. R. Chang F. Y. Pau K. Y. Wang P. S. (2003). Pharmacological effects of oxytocin on gastric emptying and intestinal transit of a non-nutritive liquid meal in female rats. *Naunyn Schmiedebergs Arch. Pharmacol.* 367 406–413. 10.1007/s00210-003-0690-y 12690433

[B295] Wu L. Meng J. Shen Q. Zhang Y. Pan S. Chen Z. (2017). Caffeine inhibits hypothalamic A1R to excite oxytocin neuron and ameliorate dietary obesity in mice. *Nat. Commun.* 8:15904. 10.1038/ncomms15904 28654087 PMC5490268

[B296] Wu Z. Xu Y. Zhu Y. Sutton A. K. Zhao R. Lowell B. B. (2012). An obligate role of oxytocin neurons in diet induced energy expenditure. *PLoS One* 7:e45167. 10.1371/journal.pone.0045167 23028821 PMC3445456

[B297] Xi D. Long C. Lai M. Casella A. O’Lear L. Kublaoui B. (2017). Ablation of oxytocin neurons causes a deficit in cold stress response. *J. Endocr. Soc.* 1 1041–1055. 10.1210/js.2017-00136 29264556 PMC5686635

[B298] Xiao L. Priest M. F. Nasenbeny J. Lu T. Kozorovitskiy Y. (2017). Biased oxytocinergic modulation of midbrain dopamine systems. *Neuron* 95 368.e5–384.e5. 10.1016/j.neuron.2017.06.003 28669546 PMC7881764

[B299] Yamashita H. Okuya S. Inenaga K. Kasai M. Uesugi S. Kannan H. (1987). Oxytocin predominantly excites putative oxytocin neurons in the rat supraoptic nucleus in vitro. *Brain Res.* 416 364–368. 10.1016/0006-8993(87)90920-6 3620965

[B300] Yamashita M. Takayanagi Y. Yoshida M. Nishimori K. Kusama M. Onaka T. (2013). Involvement of prolactin-releasing peptide in the activation of oxytocin neurones in response to food intake. *J. Neuroendocrinol.* 25 455–465. 10.1111/jne.12019 23363338 PMC3664423

[B301] Yamasue H. Yee J. R. Hurlemann R. Rilling J. K. Chen F. S. Meyer-Lindenberg A. (2012). Integrative approaches utilizing oxytocin to enhance prosocial behavior: from animal and human social behavior to autistic social dysfunction. *J. Neurosci.* 32 14109–14117. 10.1523/JNEUROSCI.3327-12.2012 23055480 PMC6622380

[B302] Yang Z. Han D. Coote J. H. (2009). Cardiac sympatho-excitatory action of PVN-spinal oxytocin neurones. *Auton. Neurosci.* 147 80–85. 10.1016/j.autneu.2009.01.013 19269259

[B303] Yao S. Bergan J. Lanjuin A. Dulac C. (2017). Oxytocin signaling in the medial amygdala is required for sex discrimination of social cues. *eLife* 6:e31373. 10.7554/eLife.31373 29231812 PMC5768418

[B304] Yi K. J. So K. H. Hata Y. Suzuki Y. Kato D. Watanabe K. (2015). The regulation of oxytocin receptor gene expression during adipogenesis. *J. Neuroendocrinol.* 27 335–342. 25702774 10.1111/jne.12268

[B305] Yoshida M. Takayanagi Y. Inoue K. Kimura T. Young L. J. Onaka T. (2009). Evidence that oxytocin exerts anxiolytic effects via oxytocin receptor expressed in serotonergic neurons in mice. *J. Neurosci.* 29 2259–2271. 10.1523/JNEUROSCI.5593-08.2009 19228979 PMC6666325

[B306] Yoshimura R. Kiyama H. Kimura T. Araki T. Maeno H. Tanizawa O. (1993). Localization of oxytocin receptor messenger ribonucleic acid in the rat brain. *Endocrinology* 133 1239–1246. 8396014 10.1210/endo.133.3.8396014

[B307] Yosten G. L. Samson W. K. (2014). Neural circuitry underlying the central hypertensive action of nesfatin-1: melanocortins, corticotropin-releasing hormone, and oxytocin. *Am. J. Physiol. Regul. Integr. Comp. Physiol.* 306 R722–R727. 10.1152/ajpregu.00396.2013 24598461 PMC4025068

[B308] Young L. J. Barrett C. E. (2015). Can oxytocin treat autism? *Science* 347 825–826. 25700501 10.1126/science.aaa8120 PMC4362686

[B309] Young W. S. III Shepard E. Amico J. Hennighausen L. Wagner K. U. LaMarca M. E. (1996). Deficiency in mouse oxytocin prevents milk ejection, but not fertility or parturition. *J. Neuroendocrinol.* 8 847–853. 8933362 10.1046/j.1365-2826.1996.05266.x

[B310] Yuan J. Zhang R. Wu R. Gu Y. Lu Y. (2020). The effects of oxytocin to rectify metabolic dysfunction in obese mice are associated with increased thermogenesis. *Mol. Cell Endocrinol.* 514:110903. 10.1016/j.mce.2020.110903 32531419

[B311] Zhang B. Qiu L. Xiao W. Ni H. Chen L. Wang F. (2021). Reconstruction of the hypothalamo-neurohypophysial system and functional dissection of magnocellular oxytocin neurons in the brain. *Neuron* 109 331.e7–346.e7. 10.1016/j.neuron.2020.10.032 33212012

[B312] Zhang G. Bai H. Zhang H. Dean C. Wu Q. Li J. (2011). Neuropeptide exocytosis involving synaptotagmin-4 and oxytocin in hypothalamic programming of body weight and energy balance. *Neuron* 69 523–535. 10.1016/j.neuron.2010.12.036 21315262 PMC4353647

[B313] Zhang G. Cai D. (2011). Circadian intervention of obesity development via resting-stage feeding manipulation or oxytocin treatment. *Am. J. Physiol. Endocrinol. Metab.* 301 E1004–E1012. 21828335 10.1152/ajpendo.00196.2011 PMC3214005

[B314] Zhang H. Wu C. Chen Q. Chen X. Xu Z. Wu J. (2013). Treatment of obesity and diabetes using oxytocin or analogs in patients and mouse models. *PLoS One* 8:e61477. 10.1371/journal.pone.0061477 23700406 PMC3658979

[B315] Zhang Z. H. Felder R. B. (2004). Melanocortin receptors mediate the excitatory effects of blood-borne murine leptin on hypothalamic paraventricular neurons in rat. *Am. J. Physiol. Regul. Integr. Comp. Physiol.* 286 R303–R310. 10.1152/ajpregu.00504.2003 14707012

[B316] Zhou F. Yu T. Du R. Fan G. Liu Y. Liu Z. (2020). Clinical course and risk factors for mortality of adult inpatients with COVID-19 in Wuhan, China: a retrospective cohort study. *Lancet* 395 1054–1062. 10.1016/S0140-6736(20)30566-3 32171076 PMC7270627

[B317] Zigman J. M. Jones J. E. Lee C. E. Saper C. B. Elmquist J. K. (2006). Expression of ghrelin receptor mRNA in the rat and the mouse brain. *J. Comp. Neurol.* 494 528–548. 16320257 10.1002/cne.20823 PMC4524499

